# Mesoporous Silica-Supported MOF Composites for CO_2_ Capture: Fabrication, Synergistic Effects, and Limitations

**DOI:** 10.3390/nano16191249

**Published:** 2026-10-02

**Authors:** Chunling Xin, Dongqi Song, Lili Liu, Xiaojing Zhou, Dan Li, Wei Zhang, Yi Yu, Xia Wang, Xishi Tai

**Affiliations:** 1 Department of Chemistry & Chemical Engineering and Environmental Engineering, Weifang University, Weifang 261061, Chinaxiawangwfu@163.com (X.W.); taixs@wfu.edu.cn (X.T.); 2 Department of Advanced Agriculture and Life Science, Weifang University, Weifang 261061, China

**Keywords:** mesoporous silica, metal–organic frameworks, CO_2_ capture, composite materials, synergistic effects

## Abstract

The continuous rise in atmospheric carbon dioxide (CO_2_) concentration poses a severe challenge to the global climate, creating an urgent need for the development of efficient, stable, and economically viable carbon-capture technologies. Metal–organic frameworks (MOFs), with their ultra-high specific surface area and tunable pore architecture, have demonstrated significant potential for CO_2_ adsorption. However, single MOF materials are typically constrained by limitations such as high mass transfer resistance, poor hydrothermal stability, and difficulties in processing their powdered form. Coupling MOF with ordered mesoporous silica (MS) to construct hierarchical MOF@MS composite materials has been proven to be an effective strategy for overcoming these shortcomings. This review systematically summarizes the recent research progress on mesoporous silica-supported MOF composite materials for CO_2_ capture. First, an architecture-based taxonomy is established in which every composite is assigned according to five decision rules—nucleation site, synthesis sequence, spatial arrangement of the two phases, interfacial bonding, and the intended role of the mesoporous silica—and six architecture classes are distinguished; systems that lack either a crystalline MOF phase or a siliceous mesoporous phase (MOF-on-MOF hybrids, wood-supported MOF, and amine-loaded silica) are explicitly excluded from the comparative analysis. Second, the principles, advantages, and drawbacks of the individual preparation routes, including in situ growth, confined growth, layer-by-layer assembly, post-synthetic assembly, selective etching, physical mixing, and macroscopic shaping, are discussed in detail and mapped onto that taxonomy. Third, the multiple synergistic adsorption mechanisms between mesoporous silica and MOF are deeply analyzed, encompassing the optimization of mass transfer by hierarchical pore structures, the enhancement of active site accessibility and stability through interfacial bonding, and the improvement of CO_2_ selectivity via functional group modification. On this basis, this review objectively examines the core challenges and limitations currently faced in this field, such as pore blockage, weak interfacial interactions, performance degradation under realistic flue-gas conditions (containing moisture and impurities), and high costs associated with large-scale preparation. Finally, to address these issues, future research directions are proposed from five perspectives: developing novel stable composite materials, optimizing green scalable synthesis processes, deepening multi-scale theoretical simulations, establishing standardized evaluation systems oriented toward realistic operating conditions, and exploring multifunctional integrated and stimuli-responsive materials. This review aims to provide focused and systematic guidance for the rational design of next-generation MOF@MS adsorbents for CO_2_ capture with high performance, low cost, and enhanced stability by elucidating the specific structure–performance mechanisms and practical limitations that are frequently overlooked in general surveys.

## 1. Introduction

**Figure 1:**
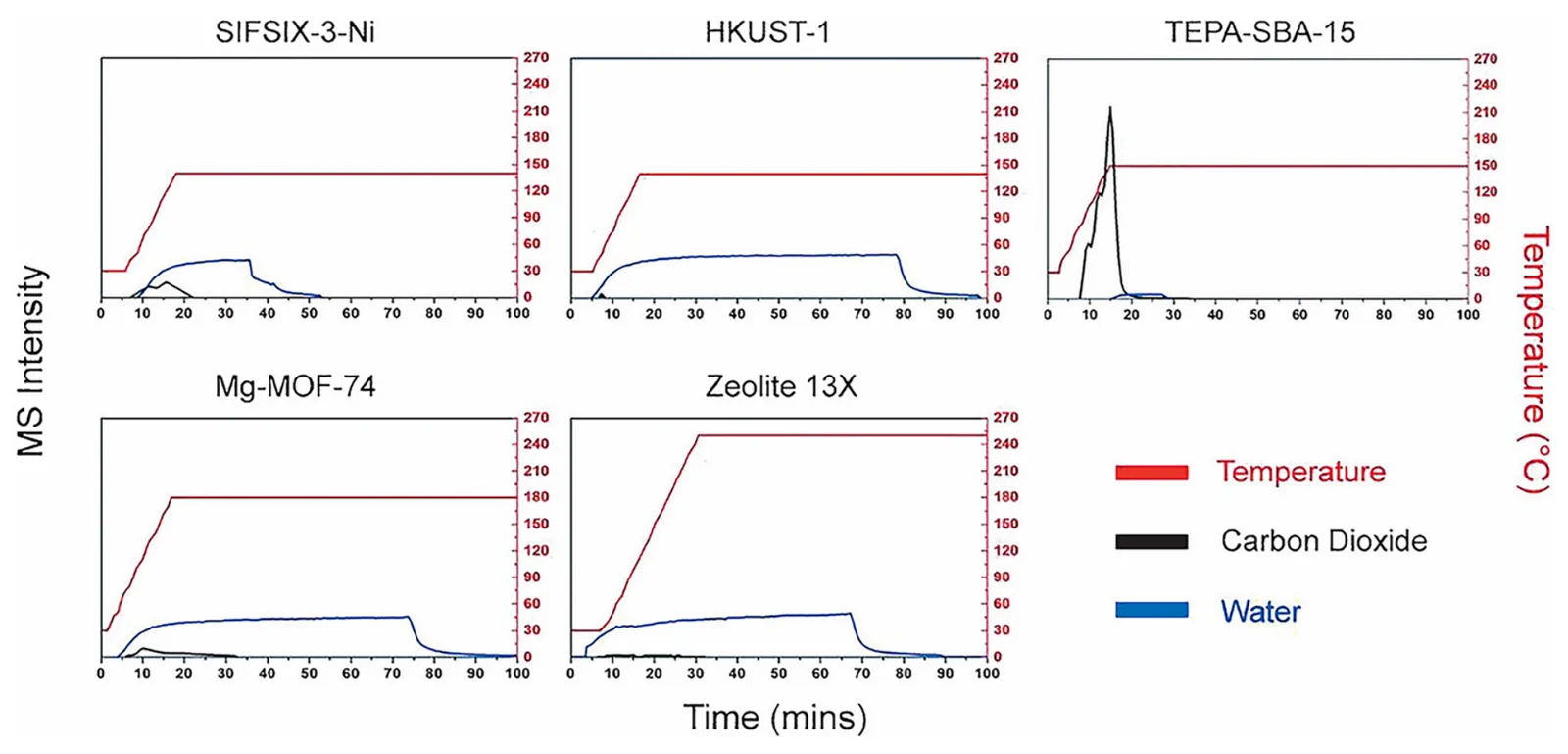
Schematic illustration of direct air capture (DAC) using physisorbent MOFs@silica composites: ultradilute CO_2_ (~400–420 ppm) is selectively adsorbed by the hierarchical pore network, and regeneration is enabled by a mild temperature/pressure swing. (Adapted from Ref. [9].) TPD plots of direct air capture of CO_2_ experiments for the five sorbents: SIFSIX-3-Ni, HKUST-1, TEPA-SBA-15, Mg-MOF-74, and Zeolite13X. The red curve is the temperature ramp profile used for desorption. The MS signals for CO_2_ and H_2_O are given by the black and blue curves, respectively.

The continuous rise in atmospheric carbon dioxide (CO_2_) concentration has become a primary driver of global climate change, posing severe challenges to ecosystems and human society. To address this crisis, carbon capture, utilization, and storage (CCUS) technologies are considered one of the key pathways to achieving global net-zero emission targets. Among various carbon-capture technologies, the adsorption method is regarded as one of the most promising solutions due to its operational simplicity, relatively low energy consumption, and environmental friendliness [1,2,3,4,5,6,7]. In recent years, with the approaching targets of the Paris Agreement, negative emission technologies such as direct air capture have also received widespread attention, whose core likewise relies on the development of efficient adsorbent materials [8]. Direct air capture represents the most demanding scenario for adsorbents because of the ultralow CO_2_ partial pressure in ambient air; as schematically summarized in Figure 1, MOF@silica composites combine high micropore density with facile mass transfer, offering a promising platform for this application.

Among numerous adsorbent materials, metal–organic frameworks (MOFs), with their ultra-high porosity, selectivity, precisely tunable pore structure, and ease of modification/functionalization, have demonstrated great potential in the field of CO_2_ capture [10,11,12,13,14,15]. The CO_2_ adsorption performance of MOF can be optimized through various strategies, including ligand functionalization, construction of open metal sites, and regulation of pore size [16]. For example, Mg-MOF-74 containing open magnesium sites exhibits excellent performance in CO_2_ separation, with a dynamic adsorption capacity of up to 8.9 wt.% and complete regeneration achievable at 80 °C [17]. However, single MOF materials still face many challenges in practical applications, such as high mass transfer resistance caused by microporous structures, slow adsorption–desorption kinetics, insufficient stability under water vapor or acidic gas environments, and difficulties in directly utilizing their powder form in industrial processes [18,19]. 

To overcome the aforementioned limitations, combining MOF with mesoporous silica (MS) materials possessing complementary properties to construct MOF@MS composites has become a highly attractive research strategy [3,18,20]. Mesoporous silica (such as SBA-15, MCM-41, KIT-6, etc.) features ordered mesoporous channels, high specific surface area, excellent hydrothermal/mechanical stability, and abundant surface silanol groups (Si-OH), making them ideal supports or structure-directing agents [21,22,23]. Furthermore, some novel mesoporous materials, such as KIT-6, exhibit superior performance in loading and mass transfer compared to traditional two-dimensional hexagonal channel materials due to their interconnected three-dimensional pore structure [24,25]. On the one hand, the interconnected channels of mesoporous silica can act as highways effectively reducing the diffusion resistance of guest molecules and enhancing the mass transfer efficiency of MOF materials [19]; on the other hand, the abundant functional groups (e.g., amino groups) on their surface can induce oriented nucleation and growth of MOF crystals, regulating the particle size, morphology, and distribution of MOF, thereby exposing more active sites [26,27]. Furthermore, the framework of mesoporous silica can provide physical support for fragile MOF crystals, enhancing the overall stability of the composite materials [28,29].

In recent years, researchers have developed various strategies for preparing MOF@MS composite materials, including in situ hydrothermal methods, layer-by-layer self-assembly, confined growth, and post-synthetic assembly approaches [18,30]. Because these routes generate architectures that differ fundamentally in interface type, mass-transfer pathway, degree of MOF dilution, and process function, this review adopts an architecture-based taxonomy with explicit decision rules (Section 2.1) instead of grouping every reported material under a single label. These composite materials often exhibit synergistic effects surpassing those of the individual components in CO_2_ capture performance [31]. For example, the MIL-101(Cr)@SBA-15 composite significantly enhanced CO_2_ adsorption capacity and adsorption rate by introducing mesoporous structures [22]; the HKUST-1@SBA-15 composite achieved simultaneous improvement in CO_2_ adsorption capacity and selectivity through amino functionalization [26]; and the CPO-27-Mg@KIT-6 composite demonstrated excellent CO_2_ affinity, even exhibiting adsorption capacities exceeding those reported in the literature under high pressure [21]. Furthermore, some novel mesoporous materials, such as KIT-6, exhibit superior performance in loading and mass transfer compared to traditional two-dimensional hexagonal channel materials due to their interconnected three-dimensional pore structure [20]. These studies indicate that the synergistic effect between MOF and mesoporous silica is not only reflected in the optimization of pore structure but also in the comprehensive enhancement of active site accessibility, adsorption thermodynamics, and resistance to environmental interference.

Despite the remarkable progress made by MOF@MS composite materials in the field of CO_2_ capture, their large-scale application still faces some inherent limitations. For instance, excessive growth or disordered accumulation of MOF within mesoporous channels may lead to pore blockage, thereby reducing the effective specific surface area of the material [32]; if the interfacial bonding between MOF and the silica support is insufficiently strong, it may cause performance degradation of the material during long-term cycling; furthermore, controllable preparation that simultaneously achieves high MOF loading, excellent dispersion, and structural stability remains a technical challenge [33,34,35]. At the same time, the cost of large-scale synthesis of materials and their long-term service performance under realistic flue-gas conditions (containing moisture and impurities) are also key bottlenecks restricting their transition from laboratory to industrial applications [36,37]. It is noteworthy that recent research has successfully fabricated monolithic MOF materials with hierarchical pore structures via 3D printing by using silica nanocages as a green binder, offering a new solution to overcome the challenges associated with powder processing [28].

Although several excellent reviews have surveyed MOF–inorganic composites and MOF-based hybrid adsorbents for CO_2_ capture, a critical gap remains in the systematic understanding of mesoporous silica (MS) as a specific support. Previous works have either covered a broad spectrum of inorganic materials (e.g., zeolites, metal oxides, and silica) with limited depth on each [30], or provided a general overview of MOF–silica composites across diverse applications, such as catalysis, drug delivery, and chromatography, where CO_2_ capture was only one of several topics [18,38]. More recently, Gebremariam et al. and Wang et al. delivered comprehensive comparisons of hybrid MOF adsorbents for carbon capture, including MOF–silica systems; however, their broad scope across various hybridizing materials (carbon, zeolites, metal oxides, etc.) inevitably limits the depth of discussion on the specific structure–performance relationships unique to MS-supported MOF [19,39].

In contrast, the present review is deliberately focused on MS-supported MOF and aims to provide a dedicated and in-depth analysis that is not achievable in broader reviews. Specifically, we address three unresolved questions that remain underexplored: (1) How do the textural properties of MS (e.g., pore symmetry, interconnectivity, and surface silanol density) dictate the growth mode and spatial distribution of MOF within confined spaces? (2) What are the mechanistic origins of synergistic effects—beyond simple pore hierarchy—particularly regarding interfacial chemical bonding and its impact on active-site accessibility and hydrothermal stability? (3) What are the practical limitations and trade-offs (e.g., pore blockage vs. loading capacity, and interfacial stability vs. synthesis complexity) that must be addressed for industrial-scale deployment?

By systematically comparing preparation strategies, evaluating the synergistic mechanisms at the molecular-to-macroscopic scale, and critically assessing performance under realistic flue-gas conditions, this review offers a decision-oriented framework for researchers and engineers. Ultimately, this review aims to bridge the gap between materials design and practical CO_2_ capture applications, guiding the development of next-generation MS-supported MOF composites with enhanced performance, stability, and scalability.

To ensure transparency, reproducibility, and systematic coverage, this review follows a structured literature search protocol. A systematic search was conducted in the Web of Science database using the terms “metal–organic framework” or “MOF” or “MOFs”, combined with “mesoporous silica” or “SBA” or “MCM”, and further combined with “CO_2_ capture” or “CO_2_ adsorption”. The search covered publications from January 2018 to June 2026, ensuring comprehensive inclusion of the entire development period for MOF–silica composite materials used in carbon dioxide capture applications. Records were retained for the comparative analysis only when the reported solid contained both a crystalline MOF phase and a siliceous mesoporous phase in direct physical contact, so that an architecture class could be assigned (Section 2.1). Studies dealing exclusively with MOF-on-MOF hybrids, with wood- or other lignocellulose-supported MOF, or with amine-functionalized silica that contains no MOF at all were kept as out-of-scope reference systems (Section 2.7) and are never used as evidence for MOF–silica synergy.

## 2. Preparation Strategies of Mesoporous Silica-Supported MOF Composites

The core of constructing efficient MOF@MS composite materials lies in how to controllably grow or assemble MOF onto mesoporous silica supports to achieve synergy in structure, interface, and function between the two components. Section 2.1 first establishes an architecture-based taxonomy together with the decision rules used for class assignment, because materials that all carry the label “MOF@MS” differ fundamentally in interface, mass-transfer pathway, dilution degree, and process function and therefore cannot be compared directly. Section 2.2, Section 2.3, Section 2.4, Section 2.5 and Section 2.6 then reorganize the preparation routes and clarify the overlap between terms that are frequently used interchangeably: in situ growth, confined growth, layer-by-layer assembly, post-synthetic modification and assembly, selective etching, physical mixing, and macroscopic shaping. Each route is mapped onto the architecture class that it produces; routes that merely transform an existing architecture, as well as routes that are precursor-delivery modes rather than architectures, are identified as such. Finally, Section 2.7 defines the scope boundaries of the MS–MOF family.

### 2.1. Architecture-Based Taxonomy and Decision Rules for MS–MOF Composites

Because the label “MOF@MS composite” is currently applied to materials that differ fundamentally in interface type, mass-transfer pathway, degree of MOF dilution, and process function, a rigorous classification is a prerequisite for any meaningful performance comparison. The same conclusion has been reached in adjacent families of MOF composites, where taxonomy-driven surveys have shown that materials reported under one single label differ in interface type, mass-transfer pathway, and process function, and that only a classification built on the interface itself supports a like-for-like comparison [38,40,41]. In this review, a mesoporous silica–MOF (MS–MOF) composite is defined as a solid in which (i) a crystalline MOF phase and (ii) an ordered or hierarchically ordered siliceous mesoporous phase coexist, and (iii) the two phases are in direct physical contact through a common interface. Each material is assigned to an architecture class using five orthogonal decision rules, applied in the following order: (1) nucleation site, that is, whether MOF nuclei form on the external silica surface, inside the mesopore void, or in the bulk solution prior to deposition; (2) synthesis sequence, that is, which phase exists first (silica-first, MOF-first, or simultaneous one-pot formation); (3) spatial relationship of the phases, that is, external surface decoration, intraporous confinement, concentric core–shell encapsulation, yolk–shell with a void gap, macroscopic interpenetration inside a shaped body, or random mechanical blending; (4) interfacial bonding, that is, non-bonded physical contact, hydrogen bonding or electrostatic attraction, metal–silanol (M–O–Si) coordination, or covalent linkage; and (5) intended role of the mesoporous silica, that is, support, nanoreactor or structure-directing agent, protective shell, binder and macropore generator, co-adsorbent, or inert diluent. A material enters the comparative analysis only when all five attributes can be assigned unambiguously; systems in which either required phase is absent are treated as out-of-scope reference systems (Section 2.7). Two of the five rules deserve particular emphasis because they are the ones most often reported in primary studies: surface silanol groups are not passive anchoring points but take part directly in the chemistry of the phase that is anchored to them [42], and the spatial distribution of a functional phase inside the pore void, rather than its total loading, governs the sorption kinetics that can actually be attained [43].

On this basis, six architecture classes are distinguished and are used consistently throughout this review (Table 1). Class I (surface-decorated, MOF-on-silica) comprises materials in which MOF crystallites nucleate at external silanol or amino sites and grow outward from the silica surface. Class II (intraporously confined, MOF-in-mesopore) comprises materials in which nucleation and growth occur inside the mesopore void, so that the crystal size is limited by the pore diameter. Class III (encapsulated core–shell, silica-on-MOF) comprises materials in which a pre-formed MOF crystal is enveloped by a continuous silica shell. Class IV (yolk–shell) is derived from Class III by partial removal of the shell or of an intermediate sacrificial layer, leaving a mobile MOF core separated from the shell by a void. Class V (silica-bonded monolith or shaped body) comprises macroscopic objects in which pre-formed MOF crystals are held together by a siliceous binder. Class VI (physical blend) comprises mechanical mixtures of the two pre-formed phases with no engineered interface; because it lacks a designed interface, Class VI is used in this review as the null-hypothesis benchmark against which a genuine synergistic effect has to be demonstrated, rather than as a composite architecture in its own right. Architectures whose asymmetry is compositional rather than interfacial, such as side-selective and Janus-type MOF composites, are consequently not introduced here as separate classes, because their defining attribute is the spatial distribution of two different functional phases within one particle and not the MOF–silica interface itself [40].

Because the primary literature uses synthesis labels inconsistently, every class assignment made in this review is decided on structural evidence rather than on the label adopted in the original publication. Three lines of evidence carry most of the diagnostic weight. First, electron microscopy: MOF nanocrystals located inside the pore channels or cavities of the support, without a separate population decorating the outer surface, define Class II, whereas crystallites that sit on the external surface and roughen the particle outline define Class I. Second, diffraction and porosimetry: In a Class II material, the ordered mesostructure of the support is retained in the X-ray diffractogram of the composite while its mesopore volume falls roughly in proportion to the volume of MOF formed inside the pores, whereas in a Class I material, the pore volume of the silica, normalized to its mass fraction, is largely preserved. Third, crystal size: Crystallite dimensions that saturate at or below the pore diameter of the host indicate intraporous confinement and therefore reflect a limit on crystal growth that only the pore wall can impose. A material reported as prepared by in situ growth is consequently not automatically Class I, and one prepared by layer-by-layer infiltration is not automatically Class II; the assignment follows the combined structural picture, and where both intraporous and external populations are documented, the dominant population determines the class.

**Table 1:** Architecture-based taxonomy of mesoporous silica–MOF composites and the decision rules used for class assignment.

Architecture Class	Nucleation Site	Synthesis Sequence	Spatial Arrangement of Phases	Interfacial Bonding	Role of Mesoporous Silica	Representative System (Ref.)
**Class I—Surface-decorated (MOF-on-silica)**	External silanol or amino sites of the silica	Silica first, and then MOF	MOF crystallites on the outer surface and at pore mouths; both phases externally accessible	M–O–Si coordination, H-bonding, or covalent grafting	Support, nucleation-directing agent, and dispersion promoter.	MIL-101(Cr)/MCM-41 [22]; HKUST-1@NH_2_-SBA-15 [26]
**Class II—Intraporously confined (MOF-in-mesopore)**	Inside the mesopore void	Silica first, and then MOF	MOF confined within channels or cavities; silica is the continuous phase	Pore-wall silanol coordination plus physical confinement	Nanoreactor, size-limiting host, and anti-leaching matrix.	ZIF-8@SBA-15 [27]; UiO-66-NH_2_@hollow silica [44]
**Class III—Encapsulated core–shell (silica-on-MOF)**	On the surface of a pre-formed MOF crystal	MOF first, and then silica	Concentric MOF core fully enclosed by a continuous silica shell	Siloxane condensation at the core–shell interface	Protective armor; hydrothermal and mechanical stabilizer.	MOF@SiO_2_ core–shell precursor [29]
**Class IV—Yolk–shell**	Inherited from the pre-formed MOF core	MOF first, then silica, and then etching	Mobile MOF core separated from the shell by a void	Point contacts only; the shell is anchored by residual necks	Size-selective protective shell and anti-sintering spacer	MOF@mesoporous SiO_2_ yolk–shell nanoreactor [29,45]
**Class V—Silica-bonded monolith/shaped body**	Not applicable; both phases pre-formed	MOF first, and silica binder added during shaping	MOF crystals dispersed in a continuous siliceous matrix at the millimeter scale	Siloxane or covalent bonding between binder and crystal surface	Binder, macropore generator, and mechanical-strength provider.	3D-printed HKUST-1 monolith [28]
**Class VI—Physical blend**	Not applicable; both phases pre-formed	Both phases independently pre-synthesized	Random juxtaposition of separate particles	Non-bonded physical contact (van der Waals)	Diluent or fluidization aid; null-hypothesis benchmark.	MCM-41/ZIF-67 physical mixture [46]

### 2.2. In Situ Growth on External Silica Surfaces

In situ growth is one of the most direct and widely used strategies for preparing MOF@MS composite materials. In this route the mesoporous silica (e.g., SBA-15, MCM-41, and KIT-6) is synthesized first and is then dispersed in a precursor solution containing metal salts and organic ligands; subsequently, through solvothermal or hydrothermal reactions, MOF crystals nucleate and grow directly on the silica surface [18,30]. The decisive attribute is the nucleation site: nuclei form at the external surface, so the resulting MOF crystallites remain accessible from the gas phase, whereas in confined growth (Section 2.3) they appear inside the pore void. Because the particle size, the pore symmetry and the surface chemistry of the siliceous host can be adjusted independently, monodisperse mesoporous silica nanoparticles have been designed rationally as hosts for bulky guests [47].

**Figure 2:**
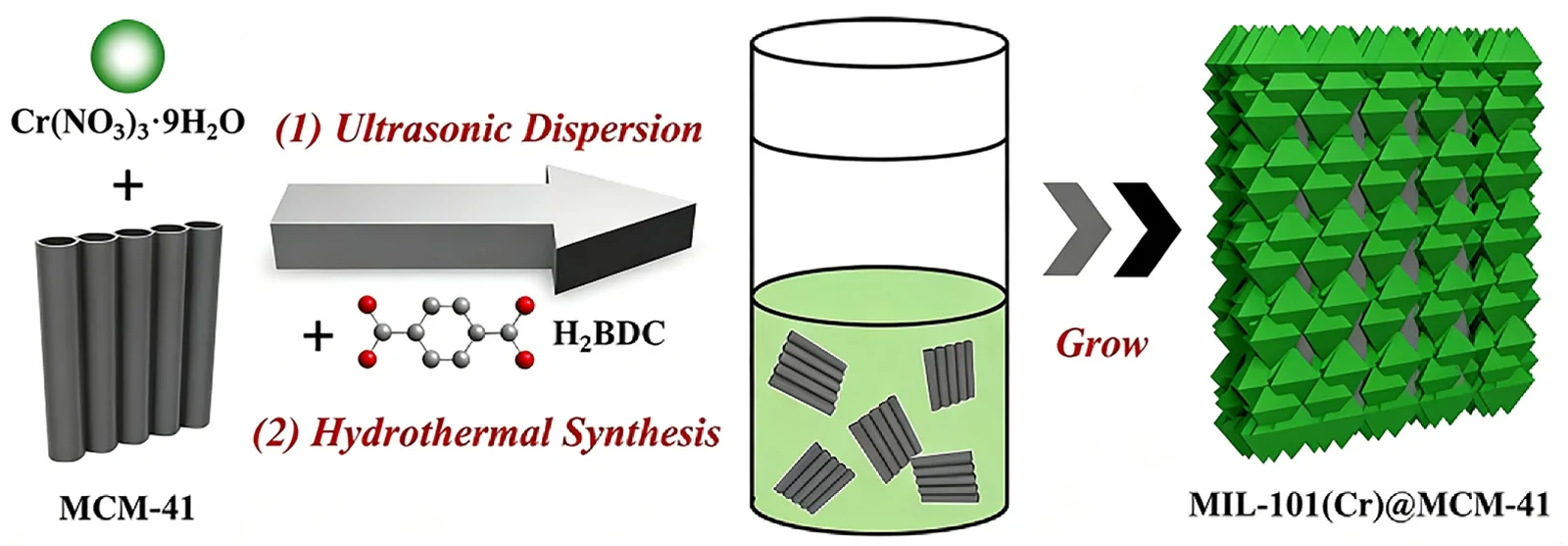
Schematic of the in situ template-directed growth of MIL-101(Cr) on mesoporous silica (e.g., MCM-41 as a structure-directing agent), yielding a layer-packed MOFs@silica composite with enhanced surface area and CO_2_ adsorption. (Adapted from Ref. [22].)

The key to the success of this method lies in the abundant silanol groups (Si-OH) on the silica surface [23]. These groups are chemically active rather than inert and assist the formation and the stabilization of the chemisorbed species on the anchored phase [42]. Silica-first assemblies built on mesocellular foam supports illustrate the same point at the level of the whole support: the texture of the siliceous host, and not only the amount of MOF deposited on it, controls both the equilibrium uptake and the uptake kinetics [48]. These silanol groups can anchor metal ions through coordination or electrostatic interactions, thereby acting as heterogeneous nucleation sites that induce preferential nucleation of MOF on the support surface rather than homogeneous nucleation in the solution. For example, one study successfully prepared a MIL-101(Cr)/mesoporous silica composite with a layer-packed structure using MCM-41 as a structure-directing agent via an in situ hydrothermal method. The introduction of MCM-41 not only regulated the growth of MIL-101(Cr) crystals but also significantly enhanced the specific surface area and microporous volume of the composite, thereby improving CO_2_ adsorption performance [24]. According to the decision rules of Section 2.1, this is a Class I material: the silica is synthesized first, nucleation takes place at external silanol sites, the MOF decorates the outer surface, the interface is a metal–silanol coordination bond, and the silica acts simultaneously as support and as structure-directing agent. In the in situ route, surface silanol groups serve as heterogeneous nucleation sites that direct MOF crystallization on the support; Figure 2 illustrates how a mesoporous silica template regulates MIL-101(Cr) growth into a layer-packed architecture that improves both textural properties and CO_2_ uptake. Growing a siliceous layer deliberately around an active phase—the silicification strategy—exploits exactly this surface chemistry and has been used to stabilize dispersed metal catalysts against sintering [49].

**Figure 3:**
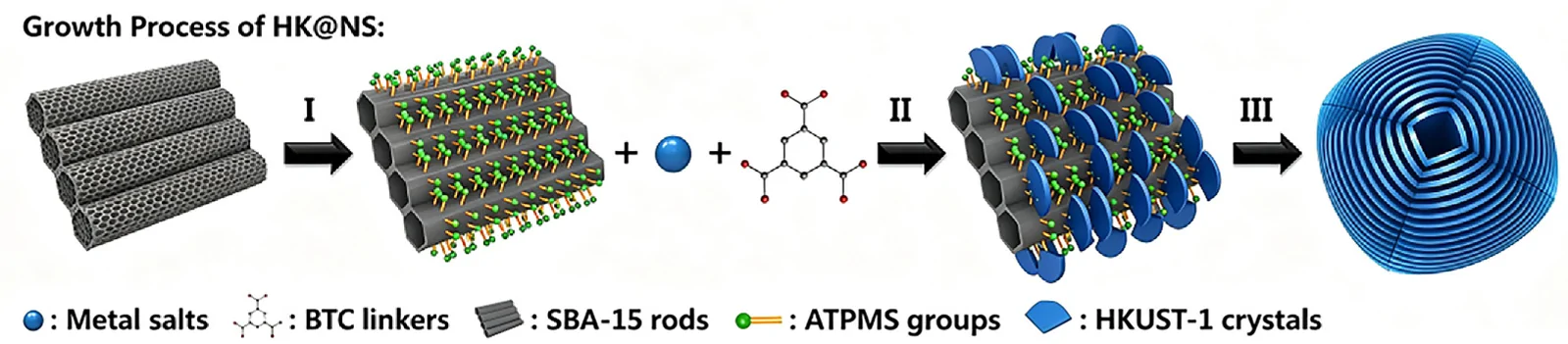
Layer-by-layer self-assembly of a hierarchical flower-like HKUST-1 composite over amino-tethered SBA-15, exposing abundant open metal sites for enhanced CO_2_ capacity and selectivity. (I) Surface functionalization, (II) amino-induced nucleation, and (III) layer-by-layer growth. (Adapted from Ref. [26].)

Furthermore, amino-functionalization of the silica support can further enhance the control over the in situ growth process [50,51,52]. For instance, in a system using amino-functionalized SBA-15 as the matrix, the amino groups can form strong coordination interactions with the metal nodes of MOFs, inducing oriented nucleation of HKUST-1 and transforming its crystallites into a hierarchical flower-like structure assembled from lamellar crystals. This structure not only increased the specific surface area and microporous volume but also exposed more metal sites, resulting in a composite material with a CO_2_ adsorption capacity 18.5% higher than that of pure HKUST-1, and a selectivity improvement of 92.6% [26]. Similarly, during the in situ growth of ZIF-8 on SBA-15, the mesoporous channels and surface silanol groups of SBA-15 effectively induced oriented nucleation of ZIF-8 through Zn^2+^ coordination and restricted its crystal size, leading to significantly enhanced catalytic activity [27]. Amino groups on the silica surface coordinate with MOF metal nodes to induce oriented nucleation, as shown in Figure 3. It should be stressed that the layer-by-layer protocol used here delivers the precursors sequentially but does not confine crystallization inside the pores: nucleation is induced at external amino sites, and the flower-like assembly grows outward from the SBA-15 surface. The material is therefore Class I (surface-decorated), which illustrates that layer-by-layer assembly is a precursor-delivery mode and not an architecture in itself (Section 2.3).

### 2.3. Confined Growth Within Mesopores and Hollow Nanoreactors

To overcome the risk of excessive MOF crystal growth and pore blockage of mesoporous channels that may occur in the in situ growth method, the confined growth strategy has been developed. This route generates Class II (intraporously confined) architectures. Its core concept is to utilize the pore channels of mesoporous silica (e.g., the 2D hexagonal channels of SBA-15 or the cavities of hollow silica) as “nanoreactors” that restrict both the diffusion of MOF precursors and the space available for crystal growth, thereby obtaining nanoscale MOF with uniform size, good dispersion, and high stability [19,53]. Confinement changes the thermodynamics, as well as the kinetics of the entrapped phase: the mobility, the reactivity, and the selectivity of a species held inside a nanoscopic void are dictated by the geometry of that void rather than by the bulk properties of the guest [43,54]. The same design logic underpins the deliberate construction of isolated metal sites and of metal or metal-hydroxide guests inside mesoporous hosts, where the size and the polarity of the confined domain, rather than the total loading, set the resulting performance [55,56]. The distinguishing attribute is, again, the nucleation site: crystallization starts inside the pore void, so the MOF becomes the dispersed phase within a continuous siliceous matrix and the silica functions as a nanoreactor and size-limiting host rather than merely as a support. Mesoporous oxide shells with an embedded metal phase, prepared without any MOF at all, provide a useful reference for the same hollow geometry and show how the accessible pore architecture, rather than the total metal loading, sets the measured performance [57].

**Figure 4:**
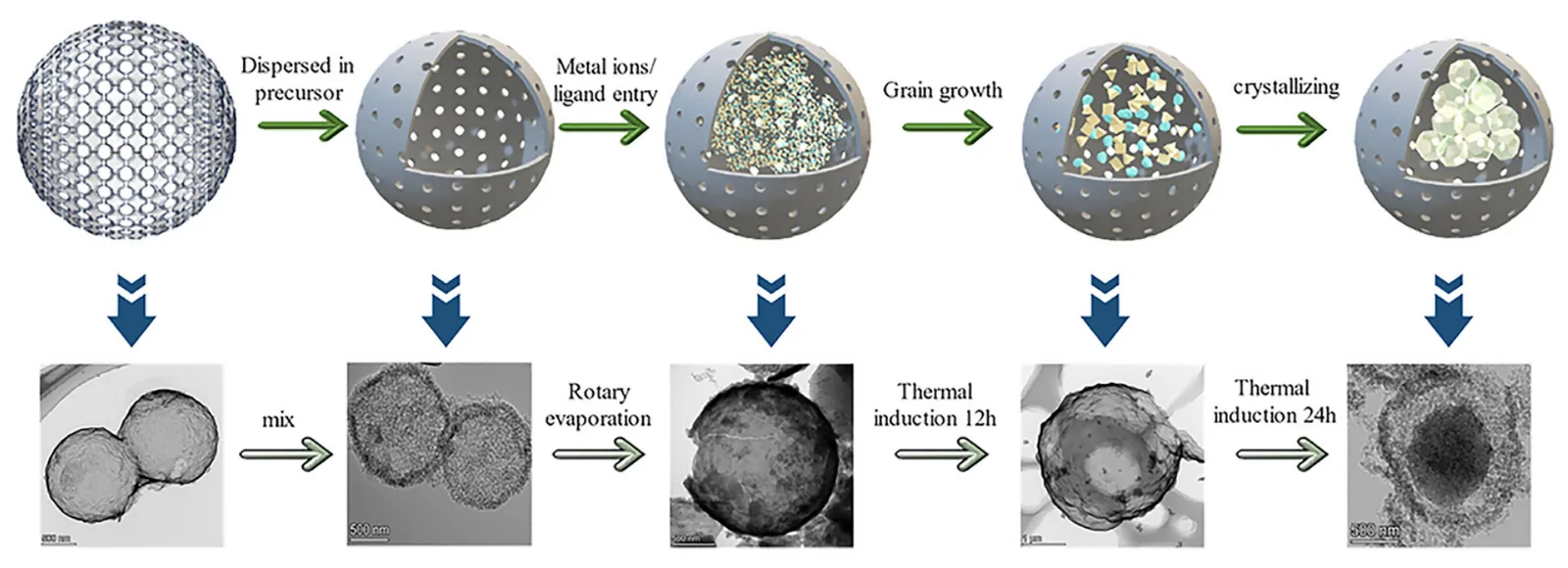
Defect-controlled confined growth of nano-MOFs within hollow silica shells (acid-modulated, e.g., benzoic acid) producing a hierarchical micro/mesoporous composite with ~48% higher CO_2_ uptake than pristine UiO-66-NH_2_. (Adapted from Ref. [44].)

Two precursor-delivery modes are commonly used, and the delivery mode must be separated from the resulting architecture. The first is layer-by-layer assembly, where the silica support is alternately immersed in metal salt solutions and organic ligand solutions so that MOF layers grow stepwise within the channels, enabling precise control over the size and loading of the MOF [27,58]. The same protocol gives a Class II material only when the solutions are infiltrated into the mesopores and nuclei form inside the channels; when nucleation is instead induced at functionalized external sites, it yields a Class I material, as in the HKUST-1/SBA-15 system described in Section 2.2. The second is vacuum-assisted impregnation, where the MOF precursor solution is drawn into the pore channels of the silica support under reduced pressure, followed by a solvothermal reaction that confines growth to that space. For example, one study employed a vacuum evaporation strategy to confine the growth of UiO-66-NH_2_ within the shell of hollow silica spheres. By introducing acid modulators such as benzoic acid to regulate structural defects, a composite material with a hierarchical micro/mesoporous structure was ultimately prepared, exhibiting a CO_2_ adsorption capacity 48% higher than that of pristine UiO-66-NH_2_ [48,59]. Introducing structural defects via acid modulators during confined growth creates additional micro/mesoporous hierarchy; Figure 4 summarizes this strategy and its ~48% CO_2_ uptake gain over the parent MOF.

Additionally, pre-loading metal oxides onto mesoporous silica and then converting them in situ into MOFs is another highly efficient confined growth strategy. For instance, a method involving the direct conversion of metal oxides supported on mesoporous silica into MOFs can efficiently prepare fluidized hybrid materials with performance comparable to bulk MOFs but with excellent abrasion resistance and fluidization characteristics, making them suitable for fluidized bed reactors [60,61,62]. This “metal oxide conversion strategy” has also been successfully applied to prepare DMSN-ZIF-8 composites, where high MOF loading can be achieved while preserving the mesoporous structure by controlling the Zn/Si ratio [33]. Because the oxide precursor is pre-loaded within the silica pore system and is converted in situ, nucleation is, again, intraporous; these materials therefore also belong to Class II, with the silica serving as nanoreactor, anti-attrition matrix, and fluidization aid.

### 2.4. Post-Synthetic Assembly of Pre-Formed Components: Physical Mixing, Chemical Grafting, and Electrostatic Adsorption

In contrast to the two bottom-up routes described above, post-synthetic strategies combine phases that have already been formed. Two operations that are frequently conflated must be distinguished here. Post-synthetic modification (PSM) strictly denotes the chemical transformation of a pre-formed framework, for example, the covalent grafting of amines onto coordinatively unsaturated metal sites or the impregnation with ionic liquids; it changes the chemistry of one phase but does not create the MOF–silica interface. Post-synthetic assembly denotes the operation that actually creates the composite, by bringing a pre-formed MOF and a pre-formed silica into contact through physical mixing, chemical grafting, or electrostatic adsorption. Only the assembly step determines the architecture class, whereas PSM is a subsequent functionalization that can be applied to any class (Section 3.4). The functionalization step is itself structure-sensitive: for aminopolymer-loaded porous hosts, the uptake and the cycling stability are governed by the defect density of the host and by the polymer–pore-wall interactions, so that one and the same nominal loading gives a different performance on different supports [63]. Impregnation with a non-volatile liquid phase, whether an ionic liquid or a deep eutectic solvent, raises the capacity of the host while simultaneously lowering its pore volume and must likewise be distinguished from the assembly step [64], as must the introduction of amino functions during framework formation rather than after it [65]. Depending on the interfacial bonding that is generated, post-synthetic assembly yields either Class VI (physical mixing, no engineered interface) or Class I and Class III materials (chemical grafting or electrostatic adsorption). Post-synthetic modification of an amino-tagged framework such as NH_2_-MIL-53(Al) is a case in point: the grafting step changes the chemistry and the catalytic behavior of the framework itself and is therefore independent of any composite architecture [66].

The main advantage of this approach is that the functionalities of both the MOF and the silica can be independently optimized before assembly. For example, one study successfully prepared an MCM-41/ZIF-67 composite by physically mixing pre-synthesized ZIF-67 with MCM-41 for aqueous phase adsorption [46]. Because no interface is engineered, this material is classified as Class VI (physical blend); it is reported here as the simplest assembly mode and, more importantly, as the null-hypothesis benchmark that has to be exceeded before a synergistic effect can be claimed (Section 3).

However, simple physical mixing often suffers from weak interfacial interactions and uneven MOF dispersion. Therefore, chemical grafting and electrostatic adsorption are more favored, because they upgrade the interface from non-bonded contact to covalent or electrostatic linkage. For instance, by covalently bonding MOF with functional groups (e.g., amino groups) on the silica support surface, or by exploiting the opposite surface charges of the two phases, the stability of the composite can be significantly enhanced. Such products are classified as Class I when the MOF decorates the silica surface and as Class III when the MOF is enveloped by silica.

### 2.5. Selective Etching and Post-Assembly Architecture Transformation

**Figure 5:**
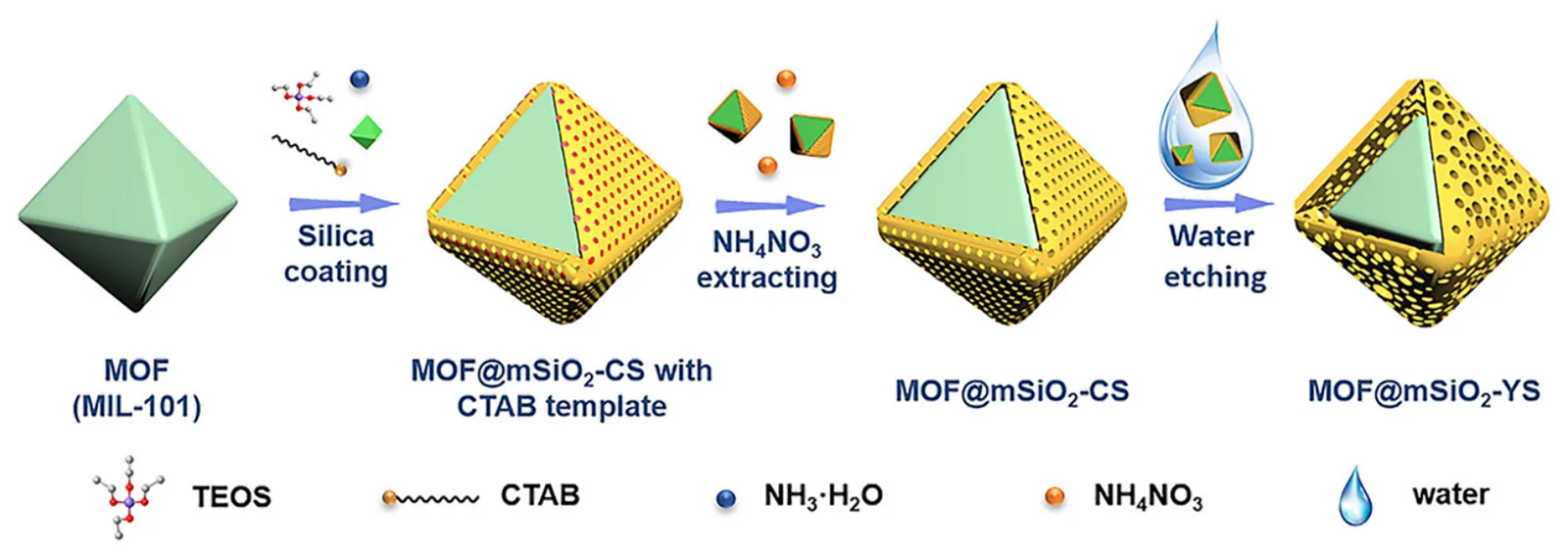
Schematic of a MOFs@mesoporous SiO_2_ yolk–shell nanoreactor fabricated by selective water etching of a core–shell precursor: the mesoporous shell protects the MOFs’ core while permitting CO_2_ transport. (Adapted from Ref. [29].) Schematic representation of the preparation of yolk–shell MOF@mSiO_2_-YS nanoreactors: Step 1, encapsulation of MOF nanocrystal by a mesoporous SiO_2_ shell; Step 2, removal of CTAB template by extraction to obtain MOF@mSiO_2_-CS; and Step 3, etching the obtained MOF@mSiO_2_-CS in hot water to obtain MOF@mSiO_2_-YS.

Selective etching is often listed alongside the routes above, but it is not an independent assembly route: it does not create a new MOF–silica interface, it transforms an architecture that already exists. Typically, a silica shell is first grown around a pre-formed MOF crystal to give a Class III core–shell solid, and part of the shell or of an intermediate sacrificial layer is then dissolved to open a void, giving a “MOF@mesoporous SiO_2_ yolk–shell nanoreactor” in which the MOF core is mobile and separated from the shell by a free volume [29,45]. Application of the decision rules of Section 2.1 gives the following: nucleation site inherited from the MOF-first synthesis; synthesis sequence MOF, then silica, and then etching; spatial relationship of a core separated from its shell by a void; interfacial bonding reduced to a small number of point contacts; and silica acting as a size-selective protective shell. This architecture not only preserves the active sites of the MOF but also provides protection through the mesoporous silica shell, significantly improving the cycling stability of the MOF. The yolk–shell architecture decouples the MOF active core from the external environment; as illustrated in Figure 5, the mesoporous silica shell preserves accessibility while markedly improving cycling stability.

### 2.6. Three-Dimensional Printing and Templated Molding

**Figure 6:**
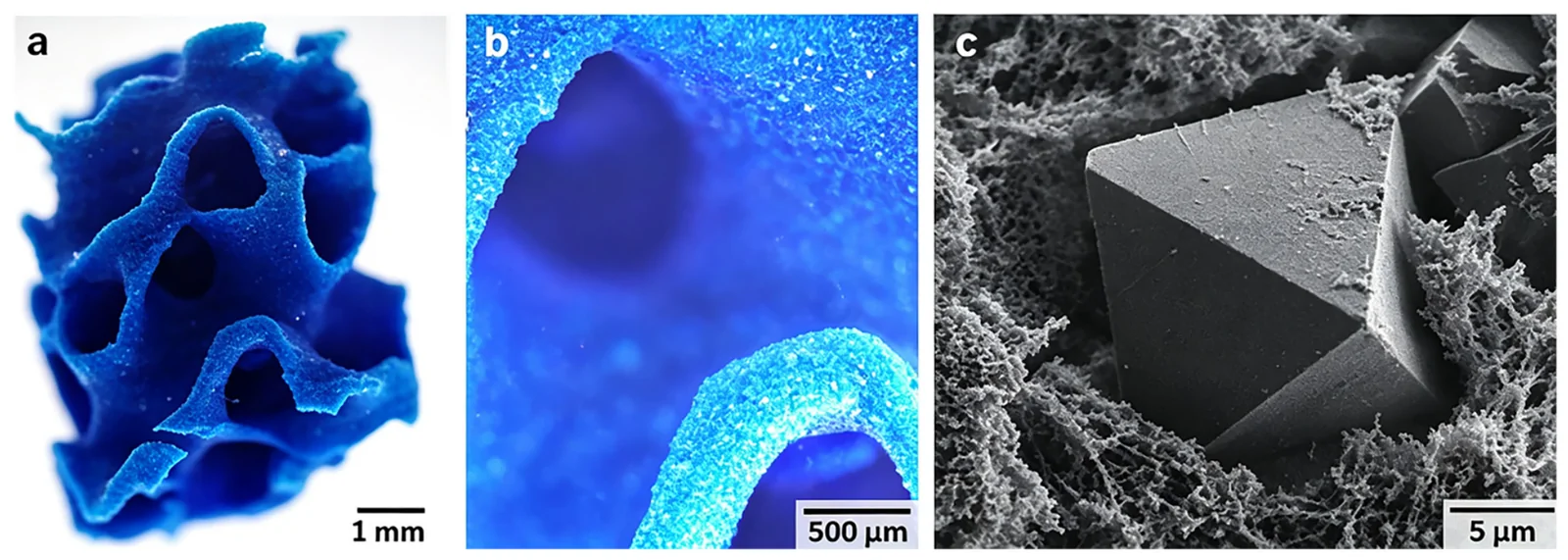
Digital light processing 3D printing of a hierarchically porous MOFs monolith using methacrylate-functionalized silica nanocages as a green binder, integrating micropores, mesopores, and macropores. (**a**,**b**) Stereomicroscope images at different magnifications of a 3D-printed MOF@SiO_2_ composite Gyroid. (**c**) SEM image of a fractured piece, showing the inside of the composite Gyroid. (Adapted from Ref. [28].)

With the development of additive manufacturing technology, 3D printing provides new ideas for the shaping of MOF@MS composite materials. One study proposed a digital light processing 3D-printing method, using methacrylate-functionalized silica nanocages as a binder to replace traditional polymers, successfully preparing hierarchical porous MOF monoliths containing HKUST-1. This approach overcomes the pore blockage issue inherent to conventional polymers; fully preserves the intrinsic microporous structure of MOF; and simultaneously introduces a hierarchical pore architecture featuring micropores, mesopores, and macropores [28,67]. Shaping is not a purely mechanical afterthought: for the low-concentration streams that motivate most of this work, the uptake that can be reached in practice is set jointly by the intrinsic site density of the porous phase and by the pressure-drop and mass-transfer characteristics of the shaped body [68,69]. According to the taxonomy of Section 2.1, this is a Class V architecture: both components are pre-formed, silica is introduced during shaping rather than during crystallization, the two phases interpenetrate at the millimeter scale, and the silica nanocages function as binder and macropore generator rather than as support or nanoreactor. Macroscopic shaping is essential to circumvent the powder-processing bottleneck; Figure 6 shows how silica-nanocage binders enable 3D-printed MOF monoliths with intact microporosity and a hierarchical pore system.

### 2.7. Scope Boundaries: Architectures Excluded from the MS–MOF Definition

Four families of materials are frequently discussed together with MS–MOF composites yet do not satisfy the definition given in Section 2.1, either because one of the two required phases is absent or because the support is not a siliceous mesoporous solid. They are consequently excluded from the comparative analysis and are cited only as reference systems. (i) MOF-on-MOF hybrids, such as ZIF-8@NH_2_-MIL-125, contain no siliceous mesoporous phase; their selectivity originates from the epitaxial junction between two frameworks and from a selection–diffusion–selection mechanism [70], and must not be invoked as mechanistic evidence for silica-containing composites (Section 3.3). (ii) Wood-supported and other lignocellulose-supported MOFs, such as Mg-MOF-74 grown on a wood sponge and coated with a hydrophobic PDMS layer [71], rely on a macroporous carbonaceous support whose surface chemistry, interfacial bonding, and mass-transfer pathway differ fundamentally from those of mesoporous silica; they belong to the biomorphic or structured-support family. (iii) Amine-loaded silicas that contain no MOF at all, such as polyethylenimine-impregnated mesoporous silica nanospheres with inverted-cone pores [72], are members of the amine–silica adsorbent family; they are used here only to illustrate how pore geometry governs amine utilization and kinetics (Section 3.1), never as evidence for MOF–silica synergy. (iv) Single-phase MOF in which the hierarchy, the interpenetration, or the ligand functionality is generated inside one single framework, such as the interpenetration isomers of amine-tagged zinc MOF [73], contain no siliceous mesoporous phase and are excluded for exactly the same reason. Recognizing these boundaries prevents the dilution effects, interface chemistries, and process functions of genuinely different material classes from being averaged into a single and potentially misleading performance comparison. Hierarchical zeolites and other crystalline microporous oxides carrying a secondary mesopore system are excluded for the same reason, because their framework composition, acidity, and interfacial chemistry are not those of an amorphous siliceous mesoporous support [74,75], and neither are silicate adsorbents that contain no crystalline MOF phase [76].

## 3. Synergistic Effects and CO_2_ Capture Performance

The core objective of compositing MOF with mesoporous silica is to leverage potential synergistic effects between the two components to overcome the inherent shortcomings of single-component adsorbents. It should be emphasized that better performance compared with one single component cannot independently confirm the existence of synergy. Reliable evaluation of synergistic behavior requires three reference benchmarks: pure MOF, pure mesoporous silica, and physical mixture with identical mass ratio, alongside mass-normalized comparison or additive-model prediction. Important trade-offs also need to be considered: introducing mesoporous silica may improve MOF dispersion and hydrothermal stability, yet gravimetric CO_2_ uptake can decline due to MOF dilution within the composite. Taking our Mg-MOF-74/MCF system as an example, the composite delivers poorer initial CO_2_ adsorption capacity than pristine Mg-MOF-74 but retains better performance after hydrothermal aging (Table 2, footnotes a and b: 1.68 mmol g^−1^ for the fresh composite against 3.70 mmol g^−1^ for pristine Mg-MOF-74 measured under the same conditions, the composite rising to 2.66 mmol g^−1^ after hydrothermal ageing against 2.39 mmol g^−1^ for identically treated pristine Mg-MOF-74; Ref. [3]); such contrasting outcomes should be described separately rather than interpreted as generalized synergy. All of the effects discussed below are interpreted with reference to the architecture class defined in Section 2.1, because the magnitude and even the sign of a given contribution depend on where the MOF nucleates; how the two phases are arranged; how strongly they are bonded; and whether the silica acts as support, nanoreactor, protective shell, binder, or diluent. The potential beneficial interactions are primarily manifested in the following aspects: optimization of pore structure and mass transfer performance, enhancement of active site accessibility and stability, and improvement of separation selectivity. Moreover, by introducing a third component such as ionic liquids, the adsorption mechanism can be synergistically regulated, thereby further enhancing the capture performance [20].

### 3.1. Synergistic Optimization of Pore Structure and Mass Transfer Performance

Single MOF materials are typically dominated by micropores. Although this is beneficial for providing high specific surface area and abundant adsorption sites, it simultaneously leads to significant diffusion resistance, resulting in slow adsorption–desorption kinetics of CO_2_ molecules. This issue becomes particularly severe under high amine loading or high humidity conditions, where pore blockage is more pronounced [18,19]. The introduction of mesoporous silica, especially materials with interconnected pore structures (such as KIT-6 and MCF), constructs a “hierarchical porous structure” in the composite material [21,77]. It should be noted that mesoporous-silica frameworks exert dual effects on mass-transfer behavior: mesoporous channels may shorten diffusion paths for guest molecules under certain structural configurations, yet deposited MOF crystals can block pore openings and bring additional interfacial mass-transfer resistance. In this structure, the micropores of MOF provide high-density adsorption sites, while the mesopores/macropores of mesoporous silica act as “molecular highways”, significantly reducing mass transfer resistance and accelerating CO_2_ diffusion rate and diffusion flux [78,79]. Importantly, a universal mass-transfer advantage cannot be generalized for all MS-MOF composite systems.

**Figure 7:**
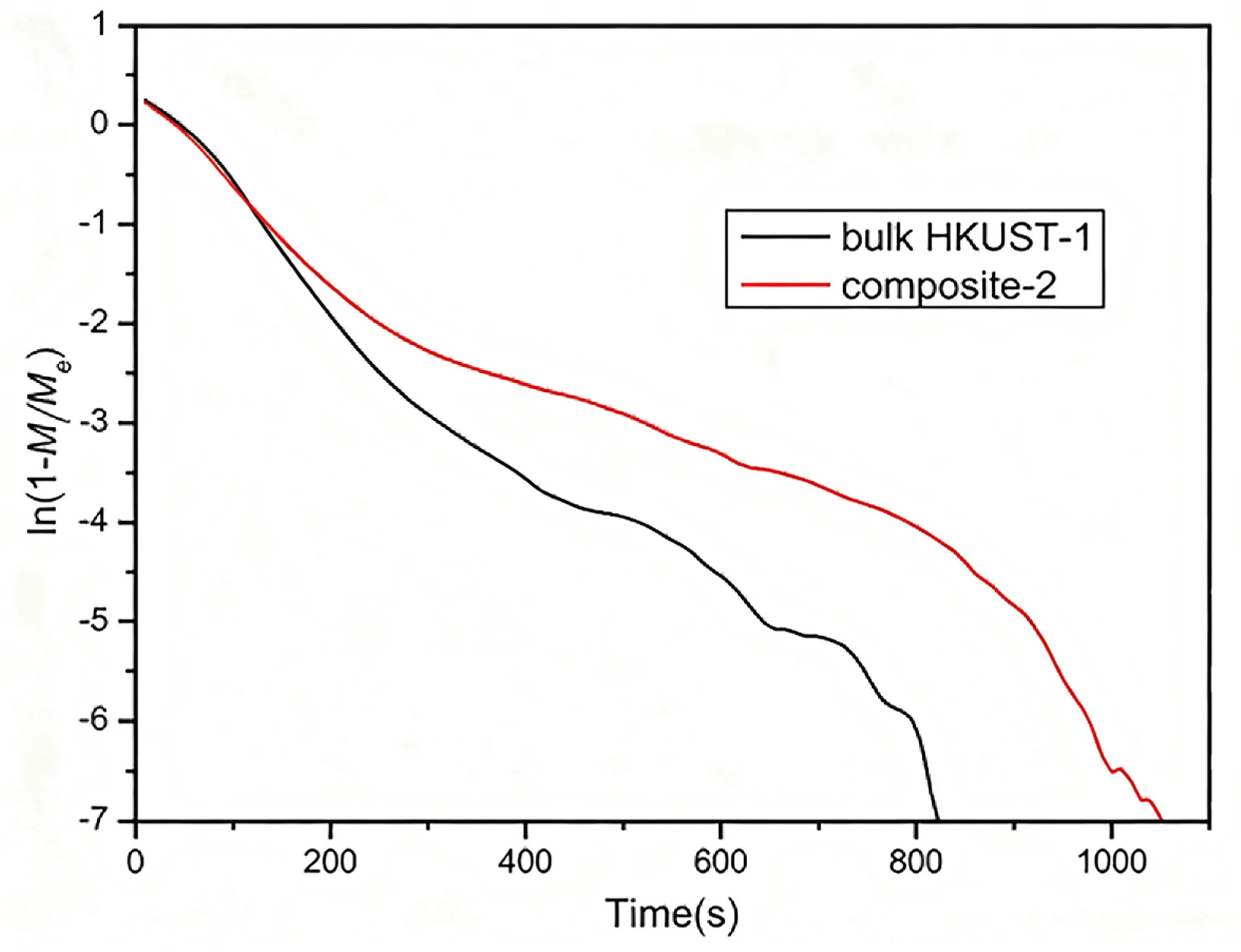
Hierarchical pore synergy in HKUST-1@siliceous mesocellular foam (MCFs): MOF micropores provide adsorption sites, while the MCF mesopores act as molecular highways, accelerating CO_2_ diffusion. CO_2_ adsorption kinetics profiles on bulk HKUST-1 and composite-2 at 298 K. (Adapted from Ref. [80].) It shows the CO_2_ adsorption kinetics profiles of composite-2 and bulk HKUST-1 at 298 K, in which the coordinate axis is ln(1 - M_t_/M_e_) and the abscissa axis is time(s). Here, M_t_ is the CO_2_ adsorption capacity at time t, and Me is the CO_2_ adsorption capacity at equilibrium. It can be found that the adsorption rate of composite-2 is higher than that of bulk HKUST-1. The adsorption kinetics may be enhanced due to an increase in molecular diffusivity in the nanoparticles, as well as the mesopores in the composites.

Experimental evidence has fully confirmed this. For example, when HKUST-1 was assembled on siliceous mesoporous cellular foams (MCFs), the resulting composite material possessed both micropores and mesopores, exhibiting superior CO_2_ adsorption capacity and adsorption rate compared to bulk HKUST-1 [80]. Similarly, in the MIL-101(Cr)/SBA-15 composite, the mesoporous structure of SBA-15 not only improved the dispersion of MIL-101(Cr) but also created more favorable mass transfer channels, resulting in significantly higher CO_2_ adsorption rate and capacity than pure MIL-101(Cr) [22,81]. This advantage of pore structure becomes even more pronounced after amine functionalization. For instance, mesoporous silica nanospheres with an inverted conical pore structure, when loaded with PEI, exhibited superior adsorption and desorption kinetics compared to materials using conventional SBA-15 as the support, demonstrating the optimizing effect of unique pore structures on mass transfer performance [70]. It should be noted that this adsorbent contains no crystalline MOF phase and therefore belongs to the amine–silica family (Section 2.7); it is quoted here only as an out-of-scope reference that illustrates how pore geometry governs amine utilization and kinetics, and not as evidence for MOF–silica synergy. Pairing MOF micropores with an interconnected mesoporous silica host creates a hierarchical network; as depicted in Figure 7, HKUST-1@MCFs exhibits superior CO_2_ capacity and rate to bulk HKUST-1 by combining high site density with low mass-transfer resistance.

**Table 2:** The CO_2_ capture performance of several representative MOF@MS composites, with the architecture class assigned according to Section 2.1.

Name	Architecture Class	MOF Loading (%)	BET Specific Surface Area (m^2^/g)	Pore Volume (cm^3^/g)	CO_2_ Adsorption Capacity (mmol/g)	Temperature (°C)	Pressure (kPa)	Feed Composition (CO_2_/N_2_ vol%)	Equilibrium Adsorption Capacity (mmol/g)	Selectivity	Adsorption Rate (mmol/g/min)	Heat of Adsorption (kj/mol)	Number of Cycles	CO_2_ Adsorption Capacity After Circulation (mmol/g)	Ref
Mg-MOF-74/MCF	II	93	655.75	0.5	1.68 ^a^	30	101	10:90	1.68	-	-	-	10	≈1.68 ^b^	[3]
UiO-66@SBA-15	II	-	393.275	0.929	1.14	40–90	101	15:85	1.14	-	-	-			[20]
UiO-66@SBA-15/[TEPA][NO_3_]	II	-	7.603	0.018	4.73	40–90	101	15:85	4.73				5	4.18	[20]
CPO-27-Mg@KIT-6 (1:1)	II	45	800	0.63	3.6	25	101	Pure CO_2_	3.6	-	-	-	-	-	[21]
CPO-27-Mg@KIT-6 (2:1)	II	65	820	0.58	5.6	25	101	-	5.6	-	-	-	-	-	[21]
MIL-101(Cr)@MCM-41	I	91.15	2843.1	1.30	2.09	25	100	15:85	2.09	8.53	-	37.03	8	2.09	[22]
HKUST-1@SBA-15	I	6.72	1782	0.76	4.93	25	101	15:85	4.93	-	2.56 × 10^−3^ 1/s	35.1~21.2	6	≈4.93	[26]
HKUST-1@Silica	V	40	1200	2.0	0.98	25	100	Pure CO_2_	0.98	-	-	50	-	-	[28]
HKUST-1@Silica	V	50	1600	1.8	1.21	25	100	Pure CO_2_	1.21	-	-	40	-	-	[28]
Cu-MOF-74@SBA-15	I	14.8~37.5	415~804	0.35~0.62	7.0~7.7	0	800	Pure CO_2_	7.0~7.7	-	-	24~26	5		[31]
Mg_2_(dobpdc)/SiO_2_	II	15.2	742	-	0.91~1.14	40	101	CO_2_:O_2_:H_2_O:N_2_ = 15:4.5:5.6:74.9	0.91~1.14	-	-	90.1	50	≈0.91~1.19	[60]
HKUST-1@MCFs	I	24.1	943	1.48	1.17	30	10	10:90	-	-	-	-	10	≈1.17	[80]
HKUST-1@MCFs	I	33.5	1057	1.17	1.40	30	10	10:90	-	-	-	-	10	≈1.40	[80]
HKUST-1@MCFs	I	41.8	1464	0.98	1.13	30	10	10:90	-	-	-	-	10	≈1.13	[80]
MIL-101(Cr)/SBA-15	I	-	1897 ± 40	1.65	3.2	30	100	Pure CO_2_	3.2	101	0.003	52–45	10	2.88	[81]

^a^ CO_2_ uptake of the fresh composite, obtained from breakthrough curves at 30 °C with a CO_2_/N_2_ = 10:90 feed at 101 kPa total pressure; pristine Mg-MOF-74 takes up 3.70 mmol g^−1^ under the same conditions (Ref. [3]). ^b^ Capacity retained after ten CO_2_ adsorption–desorption cycles; Ref. [3] reports the uptake of the composite to remain unchanged over ten cycles. The 2.66 mmol g^−1^ value quoted for this system in the same work refers to the composite after hydrothermal ageing, where it overtakes pristine Mg-MOF-74 treated identically (2.39 mmol g^−1^); it is therefore an ageing result and not a cycling result.

### 3.2. Synergistic Enhancement of Active Site Accessibility and Stability

In MOF@MS composite materials, mesoporous silica serves not only as a physical support but also actively participates in regulating the growth of MOF through its abundant surface functional groups (especially silanol groups), thereby enhancing the accessibility of active sites. On the one hand, the silanol groups or amino groups on the silica surface can anchor metal ions through coordination, inducing oriented nucleation of MOF crystals on the surface. This helps to reduce the crystallite size of MOF, exposing more metal active sites that would otherwise be encapsulated within the crystal interior [26,27,82,83]. For example, during the in situ growth of ZIF-8 on SBA-15, the confinement effect of the mesopores and the directing effect of surface functional groups successfully produced ZIF-8 nanocrystals with smaller size, exhibiting a much higher degree of active site exposure than pure ZIF-8 [27].

On the other hand, the framework of mesoporous silica can provide physical support for fragile MOF crystals, enhancing their structural stability under hydrothermal or acidic conditions. When evaluating composite stability, priority should be given to key performance indicators, including crystallinity retention, accessible pore volume, dynamic adsorption capacity, and mechanical robustness. These properties should be examined upon exposure to water vapor, O_2_, SO_x_, NO_x_, thermal cycling, and pressure-swing operating conditions. For direct-air-capture (DAC) scenarios, existing experimental datasets remain limited. We therefore analyze available low-partial-pressure (~400–420 ppm) adsorption results, humid breakthrough measurements, CO_2_-H_2_O competitive adsorption behavior, working-capacity trends, and regeneration-energy requirements. It should be explicitly highlighted that real DAC-oriented performance for most MS-MOF composites still lacks sufficient experimental validation under practical ambient-air conditions.

### 3.3. Synergistic Enhancement of Separation Selectivity

In addition to adsorption capacity, CO_2_/N_2_ selectivity is another key indicator for evaluating adsorbent performance, especially in post-combustion capture scenarios where the N_2_ content in flue gas is much higher than CO_2_. MOF@MS composite materials also demonstrate significant advantages in this aspect through synergistic effects.

On the one hand, mesoporous silica can serve as a “platform” for introducing functional groups such as amino groups that possess high affinity for CO_2_, thereby enhancing the selective adsorption of CO_2_ by the composite material [84]. For example, the composite material prepared by in situ growth of HKUST-1 on amino-functionalized SBA-15 achieved a CO_2_/N_2_ selectivity of 18.3 at 25 °C and 1.0 bar, representing a 92.6% improvement over pure HKUST-1 [26]. The reason for this enhancement is that the amino groups synergize with the open metal sites of HKUST-1, enhancing the affinity for CO_2_ while suppressing the physical adsorption of N_2_.

**Figure 8:**
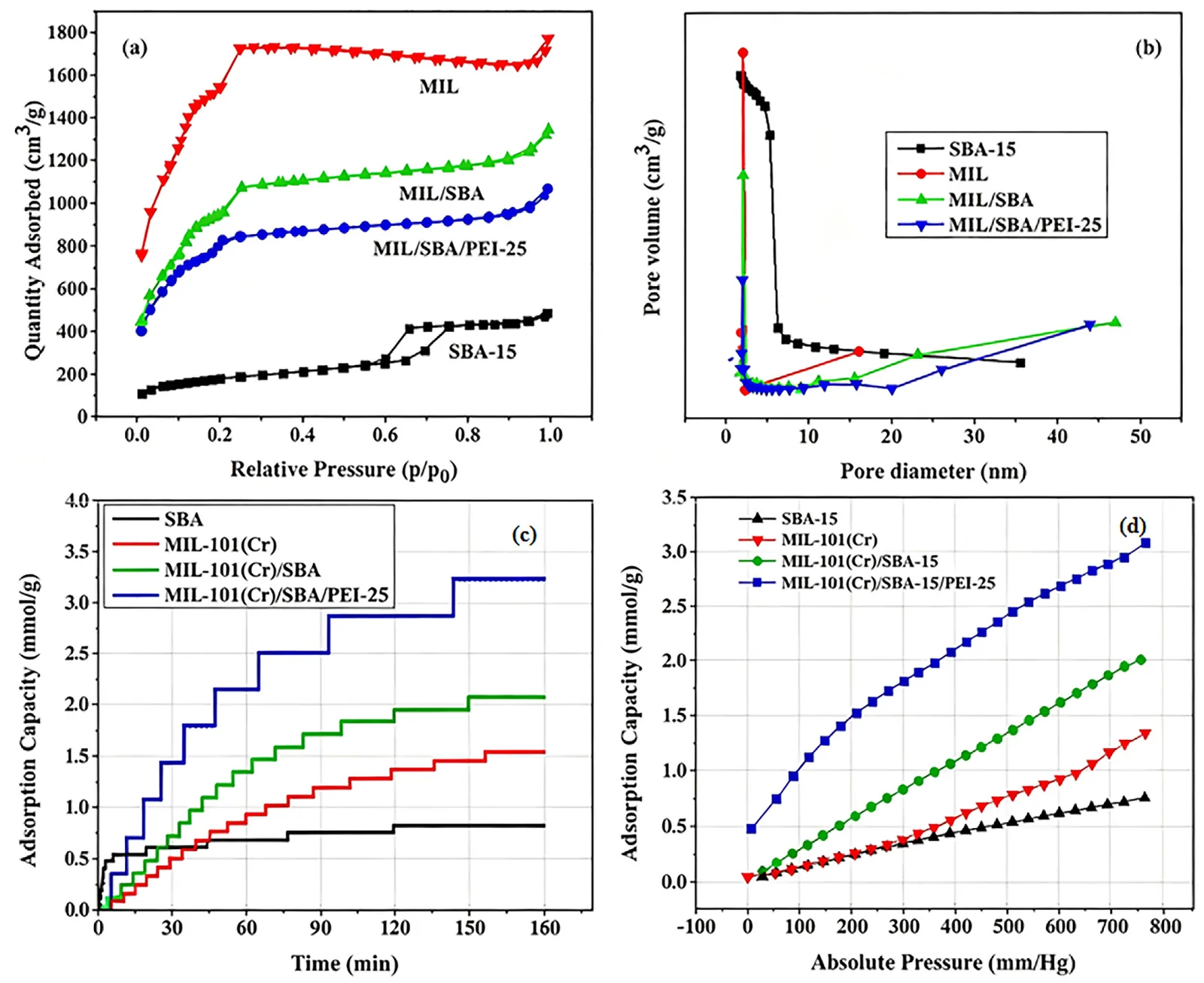
(**a**) N_2_ adsorption–desorption isotherms. (**b**) BJH pore size distributions of SBA-15, MIL-101(Cr), MIL-101(Cr)/SBA-15, MIL-101(Cr)/SBA-15/PEI-25. Pure CO_2_ adsorption capacity estimated using (**c**) TGA and (**d**) BET of SBA-15, MIL-101(Cr), MIL-101(Cr)/SBA-15, and MIL-101(Cr)/SBA-15/PEI-25. (Adapted from Ref. [81].)

On the other hand, rational pore structure design can also improve selectivity. It should be clarified that mesopores with widths of 2–50 nm cannot realize intrinsic CO_2_/N_2_ molecular-sieving effects without tailored surface chemistry. CO_2_/N_2_ selectivity should be evaluated from multiple dimensions, including pore aperture size, adsorption thermodynamics, open metal sites, surface functional groups, mixed-gas competitive experimental results, and selectivity calculation methodologies. Consistent with the scope boundaries defined in Section 2.7, MOF-on-MOF hybrids such as ZIF-8@NH_2_-MIL-125 contain no siliceous mesoporous phase; their behavior is therefore reported separately and is not taken as mechanistic evidence for MS-MOF composites, whose selectivity originates instead from the combined contribution of MOF micropores and chemically modified mesoporous silica [70]. For MOF@MS composite materials, selectivity improvements originate from the combined contributions of MOF micropores and chemically modified mesoporous silica rather than a simple two-stage physical sieving process [19]. Amine functionalization and hierarchical porosity act in concert. Figure 8 it illustrates how decorated MIL-101(Cr)/SBA-15 composites achieve cooperative enhancement of capacity and selectivity through coupled physicochemical adsorption. The CO_2_/N_2_ selectivity reaches as high as 101 for the MIL/SBA/PEI-25. Synergistic effects originate from three aspects: physical trapping within hierarchical micro-mesopores; Lewis-acid-base interactions between CO_2_ and unsaturated Cr(III) sites; and chemisorption between CO_2_ and PEI amine groups forming carbamate species. Excess PEI loading (≥50 wt%) triggers pore clogging and deteriorates CO_2_ adsorption performance [55].

### 3.4. Synergistic Regulation of Adsorption Mechanism: Ionic Liquid Modification

In recent years, incorporating ionic liquids (ILs) into MOF@MS composites has emerged as a novel synergistic regulation strategy. For instance, one study impregnated a low-volatility polyamine-based ionic liquid, [TEPA][NO_3_], into a hierarchically porous UiO-66@SBA-15 composite to fabricate a new solid adsorbent denoted as US-T60. Under simulated flue-gas conditions (15% CO_2_/85% N_2_) at 60 °C, this material achieved a remarkably high CO_2_ adsorption capacity of 4.73 mmol g^−1^, representing a 4.15-fold increase compared to the pristine UiO-66@SBA-15 composite. Kinetic and in situ characterization revealed that the system operates via a physicochemical hybrid adsorption mechanism involving two distinct CO_2_ capture pathways: the formation of ammonium carbamate ion pairs and the formation of hydrogen-bonded carbamate. This ionic liquid modification not only substantially enhanced the adsorption capacity but also endowed the material with excellent regeneration stability, retaining 93% of its capacity after five adsorption–desorption cycles [20].

### 3.5. Performance Comparison of Typical MOF@MS Composite Materials

To provide a more intuitive illustration of the above synergistic effects, Table 2 summarizes the CO_2_ capture performance of several representative MOF@MS composites. The architecture class assigned to each entry from the decision rules of Section 2.1 is also given, so that materials differing in interface, dilution degree, and process function can be compared on a like-for-like basis. Applying the structural criteria of Section 2.1, three assignments that had followed the synthesis label used in the primary papers have been corrected. Mg-MOF-74/MCF is entered as Class II and not as Class I, because electron microscopy and the retention of the MCF mesostructure show that nanosized Mg-MOF-74 particles are incorporated into the pores of the mesocellular foam instead of being deposited on its external surface (Ref. [3]). The same applies to the two entries taken from Ref. [20]: there, the UiO-66 crystals grow within the mesochannels of SBA-15 through the interaction of Zr(IV) with surface silanols, and the subsequent impregnation with [TEPA][NO_3_] is a post-synthetic functionalization that changes the chemistry of the confined MOF without relocating it, so that UiO-66@SBA-15 and UiO-66@SBA-15/[TEPA][NO_3_] both remain Class II. All the remaining entries of Table 2 were re-examined against their primary sources in the same way; no further change of class was required. In addition, the name of the second entry has been harmonized with Ref. [20] as UiO-66@SBA-15.

## 4. Challenges and Limitations

Despite the significant potential demonstrated by MOF@MS composites in CO_2_ capture, there remains a considerable journey before their large-scale industrial application can be realized. Although current research has preliminarily validated their synergistic effects, a series of urgent challenges and inherent limitations persist in areas such as material design, fabrication processes, operational performance, and economic viability.

### 4.1. Pore Blockage and Mass Transfer Resistance

**Figure 9:**
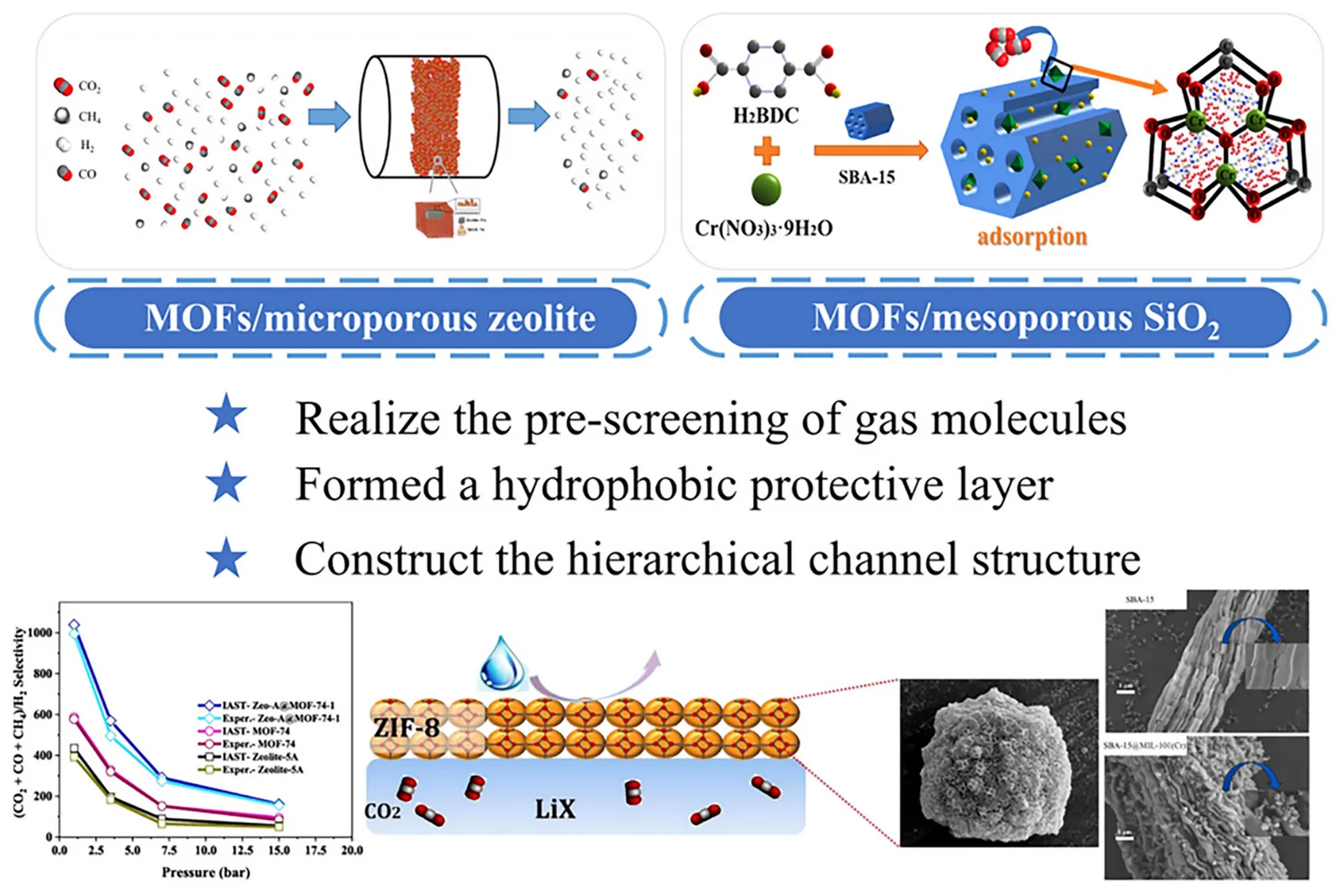
Key challenges limiting MOF@silica composites for CO_2_ capture: (i) Pore blockage by overgrown MOF crystals, (ii) weak interfacial binding causing detachment under cycling, and (iii) competitive H_2_O/acid-gas adsorption under realistic flue gas. (Adapted from Ref. [19].) MOF/porous silica CO_2_ adsorbent types and performance enhancement: (i) Realize the pre-screening of gas molecules, (ii) form a hydrophobic protective layer, and (iii) construct the hierarchical channel structure.

Although constructing hierarchical porous structures is one of the core advantages of MOF@MS composites, improper synthesis conditions in practice can lead to counterproductive results. For example, when MOF crystals overgrow within the mesoporous silica channels, they may block the pore entrances or completely fill the originally open mesopore channels. This can cause a significant decrease in the effective specific surface area and pore volume of the composite, thereby undermining the mass transfer advantages it was designed to enhance [19,85]. In some cases, excessively high MOF loading can lead to their accumulation on the external surface of the pores, forming a dense, non-porous or low-porosity layer that hinders gas molecules from entering the internal pore channels [86,87]. Furthermore, during subsequent amine functionalization modifications, if the amine loading is too high, it can also fill the pores, leading to the formation of “closed amine regions”. These amines cannot participate in CO_2_ capture, thus reducing amine utilization efficiency [88,89]. For instance, a study on amine-functionalized multi-walled carbon nanotubes showed that while the incorporation of amine groups enhanced chemisorption, excessive amine loading significantly blocked the pores and caused a reduction in overall adsorption capacity, highlighting the importance of finely tuning the loading of active components in composite systems [90]. Therefore, precisely controlling the growth location, size, and distribution of MOF within the mesopore channels, while balancing amine loading with pore porosity, is a core challenge that must be addressed when designing efficient adsorbents. Despite the demonstrated synergies, several intrinsic limitations must be overcome for practical deployment; Figure 9 summarizes the three principal challenges—pore blockage, inadequate interfacial binding, and sensitivity to moisture/impurities—that frame the future research priorities discussed below.

### 4.2. Interfacial Binding Force and Structural Stability

The interfacial binding force between MOF and silica is crucial for determining the long-term stability of the composites. Currently, most MOF@MS composites primarily bind through weak physical interactions (e.g., van der Waals forces and hydrogen bonds) or relatively weak coordination bonds (e.g., metal ions with silanol groups) [18]. This type of interfacial bonding is highly susceptible to damage during long-term cyclic adsorption–desorption processes, especially under conditions of temperature fluctuations or the presence of water vapor. This can lead to the detachment or agglomeration of MOF from the support, resulting in performance degradation [37,91]. For instance, while interfacial interactions can be enhanced through chemical grafting methods, the synthesis process is complex and may introduce harmful side reactions. Additionally, the coordination bonds within MOF themselves are fragile under humid, hot, or acidic conditions and are prone to hydrolysis, leading to structural collapse, which is particularly prominent in post-combustion flue-gas capture scenarios [10,16]. For example, Mg-MOF-74 exhibits excellent CO_2_ adsorption performance; however, it rapidly degrades in humid air, resulting in significant capacity loss, which severely limits its application in real flue-gas conditions [71]. Although some studies have employed strategies like introducing silane coatings or PDMS layers to enhance the hydrophobicity and structural stability of the materials, these strategies often increase process complexity and cost [92].

### 4.3. Influence of Water, Heat, and Impurity Gases

The real flue-gas environment is far more complex than laboratory test conditions, containing significant amounts of water vapor, sulfur oxides (SO_x_), nitrogen oxides (NO_x_), and oxygen [93]. These components pose severe challenges to the performance of MOF@MS composites. For example, water molecules exhibit strong competitive adsorption with CO_2_ at adsorption sites, which can significantly reduce the CO_2_ capture capacity of physisorbents (such as most MOF) [9]. For amine-functionalized adsorbents, while water can enhance the capacity of some amine-based adsorbents (e.g., PEI-loaded materials) by promoting the bicarbonate formation mechanism, excessive water can also lead to amine leaching or the formation of urea linkages, thereby reducing the cyclic stability of the material [94,95]. A breakthrough model study on PEI-impregnated silica indicates that under humid conditions, the presence of water alters the stoichiometry of the adsorption front by regenerating amine groups through hydrolysis; however, this also causes the adsorption front to broaden, thereby affecting dynamic adsorption performance [89].

Furthermore, MOF@MS composites typically require temperature swings during regeneration (e.g., from room temperature to 100–150 °C). Long-term high-temperature operation can lead to the volatilization or degradation of organic amine groups and irreversible collapse of the MOF framework, thereby shortening the material’s service life [96,97]. For example, for K_2_CO_3_-based adsorbents, temperatures as high as 250–300 °C may be required during regeneration to decompose the KAlCO_3_(OH)_2_ phase, which is considerably higher than the decomposition temperature of bicarbonates, thereby imposing more stringent demands on the thermal stability of the material [48].

Additionally, acidic gases like SO_x_ and NO_x_ can undergo irreversible reactions with amine groups, forming highly thermally stable sulfates or nitrates. This occupies active sites, leading to adsorbent “poisoning” and permanent deactivation. Studies have shown that, after exposure to SO_x_ and NO_x_, the CO_2_ adsorption capacity of amine-functionalized MOF decreased by 30–35% [98].

### 4.4. Scalable Fabrication and Cost Control

Transitioning from laboratory-scale gram-level synthesis to industrial-scale ton-level production presents severe scaling challenges for MOF@MS composites. Firstly, the synthesis of most MOFs relies on expensive organic ligands, toxic solvents (e.g., DMF), and high-temperature/pressure conditions, leading to prohibitively high costs [99,100,101]. In contrast, some studies have endeavored to develop fluorine-free, low-cost synthetic routes; for instance, using HNO_3_ instead of HF as a modulator for the synthesis of MIL-101(Cr) not only reduces costs and environmental hazards but also yields higher specific surface areas and adsorption performance [102]. Secondly, the reproducibility of the synthesis process is often poor, with significant performance variations between batches, making it difficult to meet the consistency requirements for industrial applications [100]. Moreover, composites are typically produced as powders, which cause significant pressure drops in industrial adsorption columns and are difficult to handle and recover, thus necessitating shaping and granulation [60]. A common process basis is, however, missing from most discussions of this transition, and its absence is exactly why cost claims are so difficult to compare. A low price for the siliceous raw material does not by itself produce a low-cost composite, because the carrier is only one term in the cost balance: surface functionalization, MOF nucleation and crystal growth, solvent consumption, washing, activation, impregnation, and final shaping each add direct expenditure, and each carry a yield penalty, so that the cost of the finished, activated, and shaped body can be dominated by the operations that follow the purchase of the oxide [99,100,103]. However, traditional shaping processes (e.g., extrusion and spray coating) often require the addition of polymeric binders. These binders can block pores and introduce inactive mass, thereby reducing the overall adsorption performance of the material [28,104]. Although some studies have explored novel shaping technologies such as 3D printing, and others have reduced costs and improved formability by growing MOF on low-cost natural minerals (e.g., halloysite nanotubes) [105], their cost-effectiveness and scalability still remain to be validated [28]. Shaping, in particular, has to be treated as a cost item in its own right rather than as a mechanical afterthought, because it fixes the bulk density, the pressure drop and the attrition resistance of the finished contactor, and thereby the vessel size and the blower duty needed for a given separation [106,107]. Structured bodies, whether monoliths or polymer/silica hollow fibers, lower the pressure drop substantially with respect to a packed bed of the same powder, yet they inevitably dilute the active phase with binder and carrier, so that part of the hydraulic gain is paid for in volumetric working capacity [107,108]. Two further quantities govern the capital rather than the materials cost and deserve to be reported routinely: the space–time yield of the composite, and the solvent inventory per unit mass of product together with the recovery fraction actually realized. Continuous and jet-based fabrication raise the former markedly with respect to batch synthesis [99,108], and for MOF UTSA-16, a continuous-flow route has been reported to reach a production rate about two orders of magnitude above the corresponding batch route [109], whereas aqueous, solvent-free or fluorine-free routes lower the latter [102]. Neither quantity is reported for the great majority of MOF@MS composites, which is the main reason why a laboratory recipe cannot, at present, be converted into a capital estimate.

### 4.5. Economic Assessment and Techno-Economic Analysis

Considering the technical challenges above, the economic viability of MOF@MS composites is the fundamental factor determining their potential for market entry. A techno-economic assessment for CO_2_ capture from steam-methane-reformer flue gas showed that even using the highest-performing MOF (IISERP MOFs2) in a vacuum swing adsorption process, the CO_2_ avoidance cost was 73.0 €/tCO_2_, which is higher than the 66.6 €/tCO_2_ for the conventional MEA (monoethanolamine) absorption technology [110]. Another study on direct air capture (DAC) indicated that at a capture scale of 100 ktCO_2_/year, the capture cost using structured monolithic adsorbents still exceeded 1500 €/tCO_2_ [111]. These data suggest that current MOF-based adsorbents do not yet have a cost advantage over mature technologies like amine scrubbing. These two figures must not, however, be read as if they belonged to a single ranking. They were obtained for different feed compositions (a few per cent CO_2_ in a reformer flue gas against about 0.04% in ambient air), for different product specifications, and for different system boundaries (capture only, against capture combined with compression and storage), and each of these choices moves the reported number by a large factor [112,113]. A comparison that places DAC, post-combustion capture, steam-methane-reformer flue gas, and MEA scrubbing side by side is therefore defensible only once the feed-gas composition, the target CO_2_ purity and recovery, and the system boundary have been harmonized; otherwise, the ranking reflects the assumptions built into each study rather than the properties of the adsorbents [110,112,114].

For this reason, the present review does not merge the published cost figures into a single number. What can be done transparently instead is to audit, parameter by parameter, how far the MOF@MS literature actually supports the inputs that a techno-economic model requires. Table 3 lists the quantities that govern processability and cost, records the extent to which each of them is reported for MOF@MS composites, and identifies where the missing information lies. The assessment is deliberately semi-quantitative: the entries are graded as reported, partly reported, or rarely reported, because, in most cases, the spread of the published values is narrower than the spread of the unstated assumptions behind them.

**Table 3:** Semi-quantitative audit of the process and cost parameters required for a like-for-like techno-economic comparison of MOF@MS composites. “Reported”, “partly reported”, and “rarely reported” refer to the MOF@MS literature surveyed in this review.

Parameter	Availability for MOF@MS Composites	What the Entry Controls, and What Is Missing
Precursor cost	Partly reported	Metal and ligand prices are usually derivable, but the cost of the mesoporous silica grade, of the grafting silane or amine, and of the solvent is rarely stated.
Solvent recovery	Rarely reported	Solvent inventory per kg of product and the recovery fraction actually achieved are almost never given, although solvent make-up can dominate the operating cost of solvothermal MOF growth [99,102].
Synthesis time	Reported	The reaction time is given, but not the total cycle time, including washing, drying, and activation, which is what fixes the reactor size.
Product yield	Partly reported	Mass yield is often replaced by the MOF loading of the composite, which does not reveal how much of the metal and ligand charged to the reactor ends up in the isolated product.
Space-time yield	Rarely reported	The one quantity that converts a recipe into a capital estimate is almost never given; continuous and jet-based routes raise it markedly [99,108].
Activation energy/duty	Rarely reported	The temperature, the duration, and the evacuation or purge conditions needed to reach the working state are described, but the corresponding energy is not quantified.
Shaping cost	Rarely reported	Binder content and the number of shaping steps are sometimes stated, but the cost of extrusion, printing, or fiber spinning is not [28,108].
Bulk density	Rarely reported	Seldom measured for the shaped body; without it, a capacity expressed per gram of composite cannot be converted into a bed volume [106].
Pressure drop	Rarely reported	Structured contactors lower it substantially with respect to packed powder, yet few MOF@MS studies report a value [107].
Mechanical and attrition strength	Partly reported	Crush strength is occasionally measured for monoliths; attrition loss under cyclic pressure or concentration swing is essentially absent [106].
Material lifetime	Partly reported	Reported as a number of cycles under dry, pure feed, rarely as an operating time under humid or impure feed [98].
Replacement rate	Rarely reported	Cannot be derived from the cycle number without a deactivation model, so the make-up cost of the sorbent is unknown.
Working capacity	Reported	Usually reported as an equilibrium uptake rather than as the difference between the loaded and the regenerated state under a defined swing [115].
Regeneration energy	Partly reported	The adsorption enthalpy is often given, but not the actual heat or vacuum duty per ton of CO_2_ captured [97].
Productivity	Rarely reported	The product of working capacity and cycle frequency, that is, ton of CO_2_ per ton of sorbent per day, is the figure a plant is sized on and is almost never reported [103].

Three conclusions follow from Table 3. First, the parameters that are routinely reported for MOF@MS composites are precisely those that characterize the material rather than the process—equilibrium capacity, selectivity, and, increasingly, cycle stability—whereas the quantities that enter a cost model directly, namely solvent recovery, space–time yield, activation duty, bulk density, pressure drop, attrition rate, and replacement interval, are reported for only a minority of systems or not at all [99,106,107]. Second, the silica component pulls in opposite directions on different entries: it lowers the precursor cost and improves the mechanical robustness, the bulk density, and the attrition resistance of the shaped body [107], but it adds at least two unit operations to the flow sheet and dilutes the active phase, so that the working capacity per unit mass of composite falls even where the capacity per unit mass of MOF does not [28]. Third, where a genuine trade-off has been quantified, it is one between working capacity and regeneration duty rather than between capacity and cost: the operating expenditure is dominated by the heat or the vacuum needed to release the CO_2_, so a higher equilibrium uptake is advantageous only if it is not bought with a proportionally higher regeneration energy [97,115]. A full numerical techno-economic analysis of MOF@MS composites is therefore not yet supportable from the published literature, and the missing entries of Table 3 are the specific measurements that would have to be supplied before it becomes so.

To promote the commercialization of MOF@MS composites, future research should focus on reducing material costs (e.g., using inexpensive metals, bio-based ligands, and natural mineral carrier); simplifying synthesis processes (e.g., room-temperature synthesis, continuous flow production, and solvent-free or aqueous-phase synthesis); improving material durability under real-world conditions (e.g., water resistance, acid resistance, and long cycle life); and developing low-energy, high-efficiency regeneration strategies [116,117]. Above all, the process parameters collected in Table 3 have to be reported together with the adsorption data, since, without them, no composite can be costed on the same basis as an established capture technology [103]. Only by achieving a balance among cost, performance, and stability can these materials truly transition from the laboratory to industrial applications.

## 5. Conclusions and Future Perspectives

In this review, we have systematically analyzed MS-supported MOF composites for CO_2_ capture, with a focused depth that complements earlier broad-scope surveys. Unlike previous reviews that covered diverse MOF–inorganic combinations or multiple applications, we have deliberately concentrated on the unique roles of mesoporous silica—particularly its pore architecture, surface chemistry, and interfacial interactions—in governing MOF growth, synergistic adsorption, and long-term stability in the field of mesoporous silica-supported MOF composites for CO_2_ capture, numerous challenges remain on the path from the laboratory to large-scale industrial application. Based on the analysis of the limitations above, future research should focus on the following key directions to promote substantial breakthroughs in this field.

### 5.1. Development of Novel and Stable Composite Systems

To overcome the bottleneck of insufficient stability of current materials under real-world conditions, future research should be dedicated to developing more robust MOF@MS composite systems. First, strengthening interfacial binding forces: By employing chemical covalent bonding strategies, such as covalently linking the ligands of MOF with the silanol groups on the silica surface, or pre-grafting functional groups on the silica surface that can coordinate with the metal nodes of MOF, the interfacial interaction between MOF and the support can be significantly enhanced. This improves the structural stability of the composite under long-term cycling and harsh environments [18,30]. Second, exploring novel MOF and support combinations: Current research largely focuses on a limited number of MOFs, such as HKUST-1, MIL-101, and ZIF-8. Future efforts should explore more MOF with ultra-high hydrothermal stability, high CO_2_ selectivity, or specific pore structures, such as the UiO-66 series, MIL-125 series, and MUF-16 [16,118,119]. For instance, MIL-120 demonstrates great promise in wet flue-gas capture applications by virtue of its outstanding water stability and low cost [120]. Concurrently, the MOF-74 family (such as Mg-MOF-74) possesses distinct advantages in capturing dilute CO_2_ due to their densely populated open metal sites, yet their inherent stability limitations necessitate mitigation through compositing strategies [121]. Concurrently, regarding supports, beyond traditional SBA-15 and MCM-41, novel mesoporous materials like dendritic mesoporous silica, silica nanocages, and periodic mesoporous organosilicas can be further investigated to achieve better pore structure tunability and functionalization potential [28,122,123]. Third, developing amorphous or hierarchical structure MOFs: Research has shown that ordered crystallinity is not a prerequisite for MOFs to perform gas adsorption functions. Amorphous hierarchical porous MOFs (e.g., NEU-2) exhibit unique advantages in CO_2_ capture due to their framework flexibility and guest-responsive capabilities [124]. Future work could explore compositing such MOFs with mesoporous silica, leveraging their unique structural characteristics to achieve more efficient capture performance.

### 5.2. Optimization of Fabrication Processes and Realization of Scalable Synthesis

Transitioning from laboratory-scale gram-level preparation to industrial-scale ton-level production is a necessary path for the commercialization of MOF@MS composites, requiring fundamental process innovation. First, developing green and low-cost synthesis routes: Exploring the use of green solvents like water or ethanol to replace toxic organic solvents, utilizing inexpensive metals (e.g., Fe and Al) or natural minerals (e.g., kaolin, halloysite) as raw materials, and employing room-temperature or microwave-assisted rapid synthesis to significantly reduce material production costs and environmental footprint [36,102,125]. As an example, the synthesis of MIL-101(Cr) via fluorine-free routes not only reduces production costs but also affords improved performance [102]. Second, promoting continuous flow synthesis technology: Traditional batch synthesis methods suffer from low efficiency and significant batch-to-batch variation. Continuous flow synthesis techniques (e.g., microfluidics and continuous stirred tank reactors) allow for precise control of reaction parameters, improving yield and reproducibility, and are key to achieving large-scale MOF manufacturing [99]. Studies have already achieved the continuous flow synthesis of MOF UTSA-16(Zn), with a production rate 77 times higher than traditional batch methods [109]. Finally, optimizing shaping processes: Avoiding traditional polymeric binders that block pores, novel shaping techniques should be developed, such as 3D printing, binder-less granulation, and electrospinning to produce aerogels. These methods can impart the required mechanical strength and shape to the materials, adapting them to the operational demands of industrial adsorption columns, while maintaining high adsorption performance [28,92,126]. Each of these options has, nevertheless, to be assessed on a common process basis that includes the additional unit operations and the associated yield losses, because a cheaper carrier or a faster synthesis does not, by itself, lower the cost of the finished composite [100,103].

### 5.3. Deepening Mechanistic Studies and Multi-Scale Simulation

Computational simulation has evolved from a purely interpretive aid into a predictive component of adsorbent design, but exploiting it for MOF@MS composites requires a clear view of what each method can and cannot resolve. At the quantum-mechanical level, DFT gives access to electronic structure, chemisorption energetics, and spectroscopic fingerprints at specific sites, yet it is confined to a few hundred atoms and picosecond timescales; it is therefore best applied to isolated motifs such as open metal sites, grafted amine pairs, or surface silanols, rather than to an entire composite particle. At the atomistic level, grand canonical Monte Carlo (GCMC) sampling samples the adsorption equilibrium of a predefined atomistic framework model; equilibrium isotherms, adsorption enthalpies, and mixture selectivities are not generated by the method itself but are subsequently calculated as ensemble averages over that sampling. This sampling requires a classical force field, in which the electrostatic contribution is commonly described by partial charges derived from DFT, whereas the remaining force-field parameters are generally taken from generic or empirically parametrized potentials and are not themselves DFT-derived. Within such a DFT-parametrized GCMC scheme, ligand functionalization can be screened computationally prior to material synthesis [127,128]. Such joint DFT–GCMC schemes have been used to rank functionalized ligands for CO_2_ uptake in MOF and to rationalize how functional groups shift both affinity and selectivity [127], whereas analogous calculations on amine-functionalized SBA-15 have clarified how different amine species and co-present flue-gas components compete for the same sites [129]. Molecular dynamics (MD) complements these equilibrium methods by resolving time-dependent behavior—self-diffusivity, pore-to-pore hopping, and the conformational dynamics of confined polymeric amines—that isotherm-based approaches cannot access [130].

Transferring this toolkit from the individual components to MOF@MS composites is, however, considerably more demanding. The silica domain is amorphous, so no unique crystallographic structure can be retrieved from a database; realistic models must instead be generated from quenched melts, carved mesopore geometries, or idealized slit and cylindrical pores, which introduce a model-dependent uncertainty that is rarely quantified. A composite further comprises at least three chemically distinct regions—the MOF crystallite, the siliceous wall, and the organic bridging or grafting layer at their interface—so a single force field has to remain balanced across inorganic; organic; and, for amine-functionalized systems, reactive species. The relevant phenomena also span several orders of magnitude in time and length: chemisorption occurs on ångström and picosecond scales, intraparticle diffusion on nanometer-to-micrometer and nanosecond-to-microsecond scales, and column breakthrough on centimeter-to-meter and second scales. Consequently, most studies to date still treat the MOF and the mesoporous silica domains separately or through simplified pore models, and genuinely interfacial simulations of MOF@MS composites remain scarce [127,130]. Quantum-accurate machine-learning potentials are beginning to relax this accuracy–cost trade-off and have recently been used to unify CO_2_ diffusion mechanisms in diamine-functionalized MOFs across timescales that neither pure DFT nor classical MD can cover alone [131].

For these reasons, simulation is most informative when embedded in a closed loop with experiment rather than used in isolation. In amine-loaded mesoporous silica, combining quasi-elastic neutron scattering and solid-state NMR with MD simulations has shown that the distribution and mobility of the confined amine phase—not merely its nominal loading—govern both sorption kinetics and the attainable CO_2_ capacity [43]. A combined experimental and simulation study of HKUST-1 grown on siliceous mesocellular foams has likewise separated the contributions of equilibrium capacity and intraparticle kinetics to the overall uptake rate, illustrating how simulation can deconvolute effects that are entangled in a measured uptake curve [132]. The same approach is indispensable for understanding humidity effects: solid-state NMR combined with computational modeling has identified the chemisorbed species formed in amine-modified sorbents and has shown that co-adsorbed water changes the dominant chemisorption pathway and stabilizes bicarbonate-type species, so models that neglect water systematically mispredict both capacity and regeneration duty [133]. The priorities set out below follow directly from the gaps identified in the preceding discussion.

A profound understanding of the CO_2_ adsorption mechanism within composite materials is fundamental for rational design and performance optimization. One approach is combining experiments with theoretical calculations. Utilizing in situ spectroscopic techniques (e.g., infrared, Raman, and solid-state NMR) combined with density functional theory (DFT) and molecular dynamics (MD) simulations can reveal, at the molecular and atomic levels, the adsorption sites, diffusion pathways, interaction mechanisms with functional groups, and the influence of water at the MOF@MS composite interface [134,135]. For example, molecular simulation studies on MIL-101(Cr) precisely revealed that CO_2_ is preferentially adsorbed in the small cages (tetrahedral cages), whereas CH_4_ tends to distribute near the C=C double bonds of benzene rings, providing an atomic-level perspective for understanding the separation mechanism of mixed gases [136]. DFT calculations have successfully revealed the key role of surface silanol groups in the CO_2_ adsorption mechanism of amine-functionalized mesoporous silica [137]. Second, developing machine-learning force fields: Traditional DFT calculations, while accurate, are computationally expensive and difficult to apply to large-scale systems. In recent years, machine learning-based force fields (e.g., moment tensor potentials and neural network potentials) can simulate the mechanical, thermal, and adsorption–diffusion behavior of MOF with accuracy approaching DFT but with significantly higher computational efficiency [138]. These tools will provide powerful assistance for high-throughput screening and design of high-performance MOF@MS composites [117]. Third, multi-scale modeling and process simulation: Establishing multi-scale coupled models, from the molecular scale to the particle scale and further to the process scale of the entire adsorption column, can systematically evaluate the impact of material properties (e.g., adsorption isotherms, kinetics, and thermal conductivity) on process performance (e.g., capture cost, energy consumption, and productivity) [139]. For example, a framework integrating molecular simulation with process simulation has been used to predict the breakthrough curves of MIL-101(Cr) for CO_2_-CH_4_-N_2_ mixtures [140].

### 5.4. Performance Evaluation Under Real-World Conditions and Standardization

The ideal conditions in the laboratory differ vastly from real industrial environments. Therefore, future research must place material performance evaluation within a framework that closely mimics practical application scenarios. First, establishing standardized testing protocols: Current research employs vastly different testing conditions (temperature, pressure, gas composition, humidity, and flow rate), making direct comparison of results difficult [141]. The academic and industrial communities should work together to establish a set of standardized testing protocols for CO_2_ capture adsorbents, particularly for specific scenarios like direct air capture and post-combustion capture, to ensure data comparability and reproducibility across different studies [101]. Such protocols should also require that every composite be assigned to an architecture class using the decision rules of Section 2.1 and that the five defining attributes (nucleation site, synthesis sequence, spatial arrangement, interfacial bonding, and function of the silica) be reported together with the adsorption data, so that materials with different dilution degrees and process functions are no longer compared directly. The same protocol should require the process parameters listed in Table 3—solvent inventory and recovery, space–time yield, bulk density, pressure drop, attrition loss, and cycle life—to be reported next to the isotherm, because, without them, two materials cannot be costed on a common basis [103]. Second, conducting long-term stability and aging studies. It is crucial to perform thousands or even tens of thousands of cyclic adsorption–desorption experiments, along with long-term aging tests lasting months, under simulated real flue-gas environments (containing humidity, SO_x_/NO_x_, and oxygen) to comprehensively evaluate material durability and performance-degradation mechanisms [91,98]. Third, integrating techno-economic analysis and life-cycle assessment. Inputting material performance data into techno-economic analysis models allows us to evaluate the total cost at different capture scales (including material cost, energy consumption, equipment depreciation, etc.) and compare it with existing technologies [110], provided that the feed-gas composition, the target CO_2_ purity and recovery, and the system boundary are harmonized before the numbers are set side by side, so that DAC, post-combustion, steam-methane-reformer, and MEA cases are not amalgamated into a single ranking [112,113] (Section 4.5). Simultaneously, a life-cycle assessment should be conducted to measure the environmental impact of the material throughout its entire lifecycle, from raw material acquisition to waste disposal, ensuring that its “carbon-negative” advantage is not negated by the high energy consumption of its preparation [142]. The results of these assessments will provide a decision-making basis for the practical application of the materials.

### 5.5. Multifunctional Integration and Intelligent Responsive Materials

Multifunctionality, in the present context, means that a single composite performs CO_2_ capture together with at least one additional function—catalysis, sensing, separation, or thermal and mechanical management—instead of acting as a passive adsorbent. MOF/SiO_2_ nanocomposites are well suited to this role because the two constituents contribute complementary attributes: the siliceous component provides mechanical robustness, tunable mesoporosity, and a versatile silanol chemistry amenable to further grafting, whereas the MOF contributes crystallographically defined active sites and molecular-level selectivity. Reflecting this versatility, MOF/SiO_2_ nanocomposites have already been developed for heterogeneous catalysis, chemical sensing, separation, and controlled release well beyond the field of carbon capture [38,143]. Porous materials that couple capture with catalytic conversion have similarly been reviewed as a distinct design class for low-concentration CO_2_ streams, in which the adsorbent determines which conversion route is thermodynamically and kinetically accessible [68]. Extending the same design logic to MOF@MS composites implies that the adsorption function should be co-designed with the functions that follow it in the process chain, rather than optimized in isolation. Luminescent frameworks that report a target analyte through a change in their emission illustrate the sensing branch of the same family, in which the sensing function is frequently combined with adsorption in one solid [144,145].

The most actively pursued combination is the coupling of capture with catalytic conversion, which is attractive because it can bypass the most energy-intensive step of the conventional capture sequence—the release and compression of pure CO_2_—by converting the captured molecule directly at or near the adsorption site. Two limiting architectures have been explored. In the first, a single bifunctional solid co-locates adsorption and catalytic sites within one pore system, so that captured CO_2_ is converted in situ into cyclic carbonates, formates or methanol; amine-grafted and dendritic mesoporous silicas are illustrative, and nanoconfinement has been shown to raise the local CO_2_ concentration and thereby the turnover of cyclic carbonate synthesis [54,146]. In the second, the two functions are spatially segregated—either in separate domains of one particle, the asymmetric or Janus-type composite design recently surveyed for high-efficiency catalytic reactions [40], or in a tandem configuration where a capture domain enriches CO_2_ for a neighboring catalytic domain. Supported metal and metal hydroxide phases exemplify the latter, hydrogenating the enriched CO_2_ under mild conditions, as demonstrated for Pd–Ni(OH)_2_ hybrids confined in SBA-15 [55,147,148,149]. The hydrogenation step itself obeys the same cooperative logic: combining surface frustrated Lewis pairs with a CuPt alloy on a supported photocatalyst raises the CO_2_ hydrogenation activity well above that of either component alone, which underlines that the conversion function has to be designed together with the capture function rather than added to it afterward [150]. Light-driven schemes extend the same principle further by combining light harvesting and CO_2_ activation within one framework [151]. Recent surveys confirm that integrated capture-and-conversion systems have become a rapidly growing design class and that adsorbent selection increasingly governs which conversion pathway is accessible [69]. MOF-derived and MOF-supported catalysts—Pd–Co obtained from MIL-53(Al), Pt–Co supported on MIL-100(Fe), Pd–Ni on MOF-derived zirconia, and mesoporous ceria derived from a Ce-MOF for CO2 methanation—show that the framework can be sacrificed or retained without losing the dispersion of the active phase [152,153,154,155].

A second axis of multifunctionality is responsiveness, that is, the ability of a material to change its pore aperture, framework conformation, or host–guest interaction reversibly under an external stimulus such as light, temperature, electric field, or pressure. Stimulus-triggered operation is attractive because it would allow the adsorption–desorption cycle to be switched on demand at the site of adsorption rather than imposed globally by a temperature or pressure swing, which is the main source of the regeneration penalty. Light is a particularly promising stimulus for this material family, since MOFs and their silica-supported composites can incorporate photoactive linkers or nanoparticles, and photo-generated charge separation is already exploited for CO_2_ reduction in MOF-based photocatalysts [151,156,157,158,159]. Translating these concepts into practical MOF@MS composites will nevertheless require that the responsive unit retain its switching fidelity over thousands of cycles in a hydrothermal environment, and that the stimulus can be delivered uniformly throughout a packed bed rather than only at its surface. The directions set out below follow directly from the foregoing analysis.

Future CO_2_ capture materials should not be limited solely to the adsorption function but should evolve toward multifunctional integration and intelligence. First, synergistic catalytic conversion: Designing MOF@MS composite materials with dual functionality for both CO_2_ capture and in situ catalytic conversion enables “capture and utilization”. For example, captured CO_2_ can be directly and catalytically converted into high-value chemicals such as cyclic carbonates, formic acid, or methanol, thereby simplifying the process and reducing overall energy consumption [2,160]. Second, developing intelligent responsive materials: Developing “smart” MOF@MS composites that respond to external stimuli such as temperature, pressure, light, or electric fields can be explored. Leveraging these responsive properties may enable precise “on–off” control of the CO_2_ adsorption–desorption process, thereby reducing regeneration energy consumption or achieving more efficient separation [161].

In summary, mesoporous silica-supported MOF composites hold great promise in the field of CO_2_ capture. However, achieving their large-scale commercial application will require sustained and collaborative innovation across multiple disciplines, including materials science, chemical engineering, and computational simulation. The focus of future research should shift from simple “performance demonstration” to solving the core triangular challenge of “scalability, stability, and economics”. Simultaneously, exploring the intelligentization and multifunctionalization of materials is essential to realizing an integrated solution for CO_2_ capture and utilization.

## Data Availability

The original contributions presented in the study are included in the article; further inquiries can be directed to the corresponding authors.
